# Early-life stress biases responding to negative feedback and increases amygdala volume and vulnerability to later-life stress

**DOI:** 10.1038/s41398-023-02385-7

**Published:** 2023-03-07

**Authors:** Ethan G. Dutcher, Laura Lopez-Cruz, E. A. Claudia Pama, Mary-Ellen Lynall, Iris C. R. Bevers, Jolyon A. Jones, Shahid Khan, Stephen J. Sawiak, Amy L. Milton, Menna R. Clatworthy, Trevor W. Robbins, Edward T. Bullmore, Jeffrey W. Dalley

**Affiliations:** 1 Department of Psychology, University of Cambridge, Cambridge, CB2 3EB UK; 2 Department of Psychiatry, University of Cambridge, Cambridge, CB2 0SZ UK; 3 Molecular Immunity Unit, MRC Laboratory of Molecular Biology, Cambridge, CB2 OQH UK; 4 Faculty of Medical Sciences, Radboud University, Nijmegen, 6525 XZ The Netherlands; 5 GlaxoSmithKline Research & Development, Stevenage, SG1 2NY UK; 6 Department of Physiology, Development, and Neuroscience, University of Cambridge, Cambridge, CB2 3EL UK; 7 Wolfson Brain Imaging Centre, Department of Clinical Neurosciences, Cambridge, CB2 0QQ UK

**Keywords:** Pathogenesis, Learning and memory, Depression, Physiology

## Abstract

Early-life stress (ELS) or adversity, particularly in the form of childhood neglect and abuse, is associated with poor mental and physical health outcomes in adulthood. However, whether these relationships are mediated by the consequences of ELS itself or by other exposures that frequently co-occur with ELS is unclear. To address this question, we carried out a longitudinal study in rats to isolate the effects of ELS on regional brain volumes and behavioral phenotypes relevant to anxiety and depression. We used the repeated maternal separation (RMS) model of chronic ELS, and conducted behavioral measurements throughout adulthood, including of probabilistic reversal learning (PRL), responding on a progressive ratio task, sucrose preference, novelty preference, novelty reactivity, and putative anxiety-like behavior on the elevated plus maze. Our behavioral assessment was combined with magnetic resonance imaging (MRI) for quantitation of regional brain volumes at three time points: immediately following RMS, young adulthood without further stress, and late adulthood with further stress. We found that RMS caused long-lasting, sexually dimorphic biased responding to negative feedback on the PRL task. RMS also slowed response time on the PRL task, but without this directly impacting task performance. RMS animals were also uniquely sensitive to a second stressor, which disproportionately impaired their performance and slowed their responding on the PRL task. MRI at the time of the adult stress revealed a larger amygdala volume in RMS animals compared with controls. These behavioral and neurobiological effects persisted well into adulthood despite a lack of effects on conventional tests of ‘depression-like’ and ‘anxiety-like’ behavior, and a lack of any evidence of anhedonia. Our findings indicate that ELS has long-lasting cognitive and neurobehavioral effects that interact with stress in adulthood and may have relevance for understanding the etiology of anxiety and depression in humans.

## Introduction

Early-life stress (ELS) in humans, particularly in the form of childhood neglect or abuse, predicts many adverse psychiatric and physical health outcomes in later life [1, 2]. Many studies, including prospective cohort studies, report a relationship between multiple types of ELS and risk of depressive and anxiety disorders in adulthood [3–5]. Further, among individuals with these disorders, meta-analytic evidence indicates that those with a history of ELS have a more severe course [6, 7]. Risk for developing a substance use disorder is also increased [8, 9], as is risk for a diverse range of physical diseases, including cardiovascular, lung, and gastrointestinal disease, as well as diabetes and autoimmune conditions [2, 10, 11].

While ELS is associated with increased risk of these adverse outcomes, none occurs deterministically following any given type of ELS [1, 12]. The associations between ELS and risk of these disorders must therefore either be causally mediated by other exposures that simply frequently co-occur with ELS (e.g., low socioeconomic status or later-life stressor exposure), or must depend on interactions between the effects of ELS and other factors, potentially including individuals’ evolving environmental circumstances throughout childhood, adolescence, and adulthood [5, 13, 14]. Years or decades often pass between ELS exposure and the onset of clear mental or physical pathology, and during that time each exposed individual navigates an enormous number of social, financial, academic, and occupational decisions as they go about their daily lives [5, 15]. If ELS biases decision making in one or more of these contexts, over time this could potentially lead to engrained patterns of cognition, emotional responding, or behavior that could eventually manifest as mental illness [16–22]. If ELS slows decision making in certain contexts, this might indirectly or directly constrain occupational achievement and increase the risk of experiencing adulthood stress [23, 24]. Predictably, due to the idiosyncratic nature of the ongoing life circumstances and events that ELS may interact with, untangling the direct causal effects of ELS itself from the effects of the subsequent decisions and exposures that those effects predispose to is exceedingly challenging to achieve through human research [13]. Research in experimental animals is essential for investigating the behavioral and neurobiological effects of ELS, because the long-term effects of ELS alone, in the absence of further psychosocial or physical challenges, or drug exposures, can be readily studied [12].

In the present study, we investigated the long-lasting effects of ELS using the repeated maternal separation (RMS) paradigm, the most widely used animal model for investigating the behavioral and physical consequences of chronic ELS [25, 26]. We used translationally relevant, objective, touchscreen-based tasks that are sensitive to disturbances in motivation for reward, sensitivity to positive and negative feedback, and decision latency that have been observed in human depression and anxiety [27–30]. We also probed for persistent anhedonia and putative anxiety-like behavior using conventional tests such as the sucrose preference test and elevated plus maze. Further, we sought to describe the short- and long-term effects of RMS on the volumes of key brain regions of possible relevance to the relationship between human ELS and subsequent depression and anxiety. Included brain regions (i.e., amygdala, hippocampus, nucleus accumbens, dorsal striatum, insula, and cingulate cortex) were selected a priori for measurement based on our ability to sufficiently delineate their borders together with reported associations between their structure or function and these human conditions [31–40]. Thus, these analyses were not driven by RMS-specific hypotheses, but instead constituted a descriptive, exploratory characterization. The exception was the amygdala, for which we hypothesized that RMS would increase volume, based in particular on prior findings of increased amygdala neuronal count and spine density [41, 42]. Given the ubiquity of stressors in human adulthood, and the role of stress in depression and anxiety etiology and pathophysiology [43, 44], we also exposed rats to a second stress during adulthood to investigate its effects on behavior and regional brain volumes.

## Methods

### Subjects

Pregnant Lister-Hooded rats (*n* = 14) were purchased from Envigo (Blackthorn, UK). Litters were delivered by spontaneous partum on gestational days 22–24. Within three days of birth, litter size was adjusted to 4–6 pups, with each litter consisting of two female and two male pups, except one litter of four males and two females. Where two litters were born within 24 h of one another, which was the case for ten litters total, pups were mixed between the litters. After litter size adjustment, litters were allocated alternately by birth time to either the maternal separation condition (*n* = 30 pups: 14 female, 16 male; reared by 7 dams) or the control condition (*n* = 28 pups: 14 female, 14 male; reared by 7 dams); > 80% power was anticipated to detect neurobiological effects [41, 42]. Post-natal day (PND) 0 was defined as the day of delivery. Food and water were available *ad libitum* until food restriction from PND 73–78 onwards (see supplementary information for detail) for behavioral testing. Body weight was measured weekly starting at PND 20, until PND 69–74 when weighing frequency increased to every 1–7 days to facilitate food restriction. Lights were always on from 21:00 to 09:00. Home cage temperature and relative humidity were maintained at 23 ± 0.2 °C and 60 ± 5% respectively. Experiments were conducted on Project License PA9FBFA9F, in accordance with the UK Animals (Scientific Procedures) Act 1986 Amendment Regulations 2012, the EU legislation on the protection of animals used for scientific purposes (Directive 2010/63/EU), and the GSK Policy on the Care, Welfare and Treatment of Animal, following ethical review by the University of Cambridge Animal Welfare and Ethical Review Body (AWERB).

### Manipulations

From PND 5 through PND 19 inclusive, pups from RMS litters were separated from their dam for 6 h a day, beginning at 11:00–12:30. During separation, dams remained in their home cages while pups were taken to a different room and placed together in an unlidded standard mouse cage inside a ventilated cabinet. One centimeter of bedding was provided and the temperature at the surface of the bedding was kept between 30 °C and 35 °C through warming of the air and use of an electric heat pad. Control pups were subject only to normal animal facility rearing, including once-weekly cage changes, which RMS animals also received. Following RMS, on PND 20, a subset of animals underwent anesthesia for sublingual blood collection (for immunological profiling; not reported here) and an MRI scan. Pups from both groups were weaned and housed in same-sex pairs at PND 20 and left undisturbed until adulthood except for weighing, once-weekly cage changes, and a brief anesthetic (duration 2–5 min) for sublingual blood collection at PND 53-55 (samples not used for any analyses presented here). Anesthesia was achieved using 3–5% isoflurane for induction and 1–2% for maintenance.

All RMS and control animals underwent a novel subchronic footshock stress in late adulthood, beginning at PND 260-304 (i.e., 8.5–10 months of age). Across 19 calendar days (referred to as stress days 0–18), on 14–16 individual days, animals were placed inside operant chambers for one 30 min session each day, during which they received up to 2 footshocks at unpredictable times. All animals received exactly 20 shocks in total. Further detail on the adult stress is provided in the supplementary information, along with a timeline of all manipulations.

### Behavioral testing

Once animals reached adulthood, behavioral testing on the following tasks was conducted: elevated plus maze (EPM; PND 66-69), novelty reactivity test (NRT; PND 70-73), and novelty preference test (NPT; PND 74-76). Measures calculated for analysis were the proportion of time spent in the open arms on the EPM [45], the total distance moved during the NRT [46, 47], and the proportion of time spent in the novel chamber on the NPT [48]. Further detail regarding all behavioral tests can be found in the supplementary information.

Two tasks were administered using touchscreen-equipped operant chambers: progressive ratio (PR) schedules of reinforcement, and probabilistic reversal learning (PRL). For both tasks, a set of 8 identical operant chambers were used (Med Associates, St. Albans, VT, USA), with a given chamber only ever being used for animals of a single sex. In both tasks, an electronic pellet dispenser delivered small 50% sucrose pellets (TestDiet, St. Louis, MO, USA) into the pellet receptacle as rewards for completing trials on the touchscreen.

The progressive ratio task was used to assess motivation for food reward [49]. Training commenced at PND 80-84, and testing concluded at PND 115-150. During each training or test session, animals were able to touch a single white square stimulus on the touchscreen a certain number of times to earn a pellet reward. After successful completion of three training stages, animals completed 9 sessions on each of 3 motivationally demanding PR schedules: PR 4, PR 8, and PR 16. In PR schedules, the number of responses necessary to earn a reward begins at 1 each session and then increases by the specified number each time a reward is earned (for example, for PR 4, the number of responses necessary to earn a reward increases as follows: 1, 5, 9, 13, 17, 21, etc.). All training and test sessions lasted for 45 min unless terminated early because 100 pellets were earned or, on PR schedules only, the animal ceased touching the stimulus for 180 s. The outcome of interest was the breakpoint per session, defined as the number of stimulus touches made during the last successfully completed trial, reflecting the maximum effort the animal was willing to expend for reward [49].

The sucrose preference test (SPT), a putative measure of anhedonia that is sensitive in the short-term to chronic high-intensity stress in animals [50, 51], was conducted twice: first before the adult stress, starting at PND 143-161, and second during the adult stress, on stress day 16 or 17 (PND 276-321). Following a habituation procedure, 4-hour tests were conducted in which animals could freely drink from a bottle containing a sucrose solution and a bottle containing water. Testing before adult stress measured preference for 0.5%, 1%, and 2% sucrose solutions, while testing during adult stress measured preference only for a 1% sucrose solution.

The probabilistic reversal learning task was used to measure multiple aspects of reward- and negative feedback-associated behavior [52–54]. Training commenced on PND 241-277 and testing continued until adult stress day 6 or 11 (PND 266-305). After successful completion of three training stages, animals were tested on the PRL task. On each trial, two visually identical stimuli (a white square overlayed with a thick black ‘X’) were presented on the left and right sides of the touchscreen. At any given time, one of these stimuli was the ‘correct’ (80% rewarded, 20% non-rewarded) stimulus and one was the ‘incorrect’ (20% rewarded, 80% non-rewarded) stimulus. If reward was triggered, a 0.5 s tone was generated, the pellet receptacle light was turned on, and a sucrose pellet was delivered. If negative feedback (non-reward) was triggered, the house light was turned on for 5 s and then the receptacle light was turned on. Following either outcome, the receptacle light remained on until the animal made a head entry into the receptacle, at which point the trial concluded and a 5 s inter-trial interval (ITI) began. If an animal touched the correct stimulus eight trials in a row (suggesting that it had successfully identified the correct stimulus), a reversal occurred, meaning that the correct stimulus became the incorrect stimulus and vice versa. Sessions concluded after either 40 min had passed, or 200 trials were completed.

Several metrics of interest were calculated from the PRL data and analyzed. The proportion of trials on which the correct target was selected was calculated to capture differences in task performance, trial count and mean latency between reward signaling onset and reward collection (“latency to collect”) were recorded to capture differences in motivation for reward and locomotor activity, and mean latency between stimulus presentation and stimulus selection (“latency to respond”) was calculated to capture differences in processing speed and attention. Additionally, indices of sensitivity to positive and negative feedback were calculated, where sensitivity was defined as the influence of a specific outcome of a decision on future decision making. To capture sensitivity to reward (or to “positive feedback”), the proportion of trial pairs where the animal first selected the correct target and was rewarded, in which they then selected the correct target again (i.e., “stayed” on the first-selected target), was calculated (the “correct-win stay proportion”). The background of a correct-win represents a strong recent reward history on the correct target, so by definition, animals that select the correct target again are more sensitive to reward than those who shift to the incorrect target. To capture sensitivity to the negative outcome (or to “negative feedback”), the proportion of trial pairs where the animal first selected the incorrect target and received signaled non-reward, in which they then selected the correct target (i.e., “switched” to the target not selected first) was calculated (the “incorrect-loss shift proportion”). The background of an incorrect-loss represents a strong recent history of signaled non-reward on the incorrect target, so animals that select the incorrect target again are less sensitive to negative feedback than those who shift to the correct target.

### Magnetic resonance imaging

Rats underwent MRI scanning for regional volumetry at three time points: PND 20 (immediately following sublingual blood collection, under a continuous anesthetic), PND 62 (median, range 61–62), and PND 285 (median, range 271–309; median days of stress before scan day: 11, range 9–13). MRI images were acquired using a 9.4 Tesla (T) horizontal bore MRI scanner (BioSpec 94/20, Bruker, Coventry, UK). The volumes of six regions of interest (ROIs) were then quantified. Detail regarding the conduct of the MRI scans, image acquisition, and image analysis can be found in the supplementary information.

### Data analysis

Data processing, graphing, and statistical analysis were performed using R v4.1.3. All analyses initially involved fitting a linear model. In situations involving repeated measures, a mixed-effects model was fit, otherwise a fixed-effects model was fit. Parametric methods were used for analyses of arena-based behavioral testing (EPM, NPT, and NRT), adult stress sucrose preference, and predicting session-level task performance from correct-win stay proportion and incorrect-loss shift proportion. Nonparametric methods (permutation testing ± bootstrapping) were used in all other cases.

Test statistics and *p*-values were provided in the main text for all significant (*p* < 0.05) and trend (*p* < 0.1) effects involving group (i.e., main effects or post-hoc effects of group, or interaction effects involving group), except where a higher-order effect (interaction term) was significant, in which case lower-order effects (interaction or main effect terms) were not reported in the main text. Test statistics and p-values not reported in the main text can be found in the supplementary information. In reporting permutation test results, test statistics and degrees of freedom derived from the unpermuted data were reported together with the p-value derived from the permutation distribution. All visualizations in the main text, except for box plots, represent estimated marginal means (EMMs) ± the standard error of the mean (SEM).

## Results

### RMS did not affect acquisition of PR or PRL tasks

Before commencing the touchscreen tasks (PR and PRL), animals progressed through a series of training tasks. The training tasks that preceded PR were fixed ratio 1 (FR1) and fixed ratio 5 (FR5); there were no significant differences between groups in the number of sessions required to complete these tasks (Fig. S2). The PRL task was preceded by touch training A (TTA), touch training B (TTB), and deterministic reversal learning (DRL). There were no significant differences between RMS and control animals in the number of sessions taken to complete TTB or DRL. For TTA, all animals except two control females required only one session (Fig. S3). There was a trend (F_1,45_ = 3.06, *p* = 0.089) difference in the number of PRL sessions completed by the time of adult stress onset, with RMS animals having slightly more experience on the task, at 15.3 ± 0.9 sessions compared to 12.8 ± 0.9 sessions for controls (Fig. S3).

### ELS slowed responding on the PRL task in adulthood, with exacerbation by further stress

In both the pre- and post-stress PRL analyses, the effect of RMS on the following behavioral parameters was examined: overall task performance (correct target selection), trial count, latency to respond on a stimulus, latency to collect reward, incorrect-loss shift proportion, correct-loss shift proportion, incorrect-win stay proportion, correct-win stay proportion, number of reversals, and perseverations per reversal. Post-stress analyses were intended to test for differential responses to adult stress by RMS and control animals, so all post-stress test and descriptive statistics were adjusted for any influence of baseline [55, 56].

**Fig. 1:**
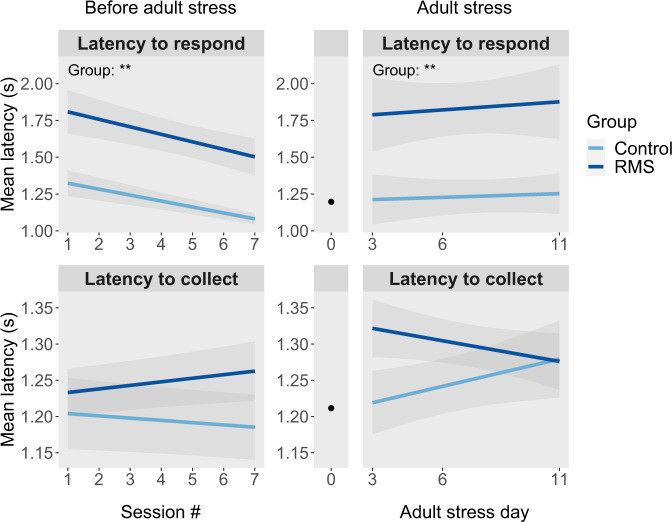
Repeated maternal separation (RMS) caused a long-lasting slowing of decision making on the PRL task, with adult stress exacerbating this effect. Animals were tested in adulthood on the spatial probabilistic reversal learning (PRL) paradigm, in which they were presented with two visually identical stimuli on a touchscreen and had to select one to respond on. The “correct” target resulted in reward 80% of the time and signaled non-reward (i.e., negative feedback) 20% of the time, while these probabilities were inverted for the “incorrect” target. After 8 consecutive responses on the correct target, the correct and incorrect targets were reversed. The number of reversals completed per session was typically between 2 and 5 and was unaffected by RMS. Left panels: In adulthood, across their first seven PRL sessions, RMS animals (*n* = 24) were consistently slower than controls (*n* = 24) to respond on one of the two stimuli, while exhibiting a similar latency to collect reward as controls. Right panels: Animals underwent daily PRL testing until commencement of the adult stress, after which they were tested on two (RMS *n* = 24–25, control *n* = 23) or three (RMS *n* = 18, control *n* = 16) further sessions. Each animal’s median performance across the final five sessions before commencement of adult stress was taken as its baseline. The black dots at stress day 0 represent the mean across all animals at baseline, with the lack of error bars signifying that mean-centered baseline is covaried for in the right panel, and that differences in regression lines thus represent differential effects of adult stress between groups. Adult stress increased latency to choose between stimuli in RMS animals significantly more than it did for controls, but had no differential effect on latency to collect reward.

Regarding the latency measures, RMS had robust effects on the latency to make a stimulus choice, but not on the latency to collect reward. Across the first seven PRL sessions, in the initial mixed-effects model involving the three main terms (group, sex, and session) plus full interactions, RMS had a significant main effect on latency to respond (F_1,45.0_ = 10.00, *p* = 0.003) in the absence of any interaction with session or sex (Fig. 1). Specifically, RMS increased the average time animals took to choose between the two stimuli by ~450 ms, from 1.20 ± 0.1 s to 1.65 ± 0.1 s, and this difference was consistent across sessions, even as both groups became quicker at responding with time. The adult stress had a differential effect on RMS vs. control animals, slowing decision making by ~400 ms in RMS animals compared to controls (F_1,41.7_ = 8.83, *p* = 0.004), with RMS animals taking 1.69 ± 0.12 seconds to decide compared to 1.29 ± 0.08 s, after adjustment for baseline differences (Fig. 1).

### ELS-exposed animals respond differentially to negative feedback in adulthood

**Fig. 2:**
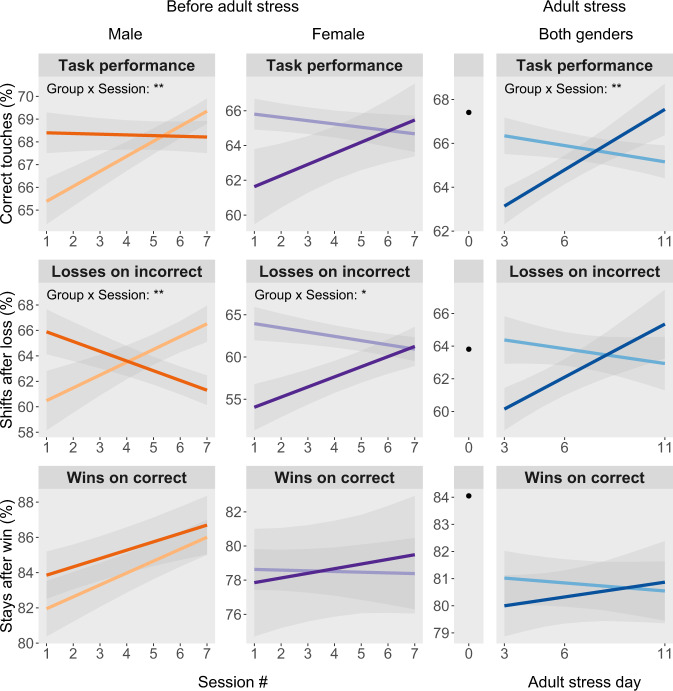
Repeated maternal separation (RMS) had long lasting effects on negative feedback sensitivity but not reward sensitivity on the probabilistic reversal learning (PRL) task, resulting in alterations to task performance. RMS males (*n* = 15, dark orange) differed from controls (*n* = 15, light orange) in their trajectories over time in both task performance (correct touch proportion) and incorrect-loss shift proportion. Follow-up testing revealed that in the first of the seven initial probabilistic reversal learning (PRL) sessions, RMS males selected the correct target more often than controls. This appeared to be driven by the tendency of RMS males to initially respond more appropriately to repeated negative feedback than controls, with a trend at session one for greater shifting to the correct (80% rewarded) target in the context of having selected the incorrect (80% non-rewarded) target on the previous trial and having received signaled non-reward (i.e., a ‘loss’). RMS females (*n* = 9, dark purple) differed from control females (*n* = 10, light purple) in their trajectory over time of incorrect-loss shift proportion, with a corresponding trend for a difference in the trajectory of task performance. Follow-up testing revealed that RMS females initially responded less appropriately to repeated negative feedback than controls, shifting significantly less after an incorrect-loss (i.e., having selected the incorrect target and received signaled non-reward). Adult stress specifically differentially affected the same metrics that were affected by RMS itself: following application of the adult stress, there was a significant group × stress day effect on task performance and a trend for a group × stress day effect on incorrect-loss shift proportion. The former interaction revealed a significant effect of group at day 3 of stress, with RMS animals (dark blue) exhibiting greater adult stress-induced impairment in task performance than controls (light blue). Black dots at stress day 0 represent the mean across all animals; the lack of error bars signifies how baseline-associated variability is removed from the adult stress analyses by including mean-centered baseline as a covariate. At stress days 3–6: RMS *n* = 24–25, control *n* = 23. At stress day 11: RMS *n* = 18, control *n* = 16.

Regarding the non-latency outcomes before adult stress (Fig. 2), the group × sex × session interaction term was significant for two response variables: correct target selection (F_1,284_ = 11.28, *p* = 0.001) and shift proportion following incorrect losses (F_1,284_ = 15.24, *p* = 0.0001). No further significant or trend effects involving group were identified. Each three-way interaction was followed up by constructing a separate mixed-effects model for each sex.

The female-specific models revealed a significant group × session interaction on shifting following a loss on the incorrect target (F_1,112_ = 6.25, *p* = 0.016), and a trend for a group × session interaction on overall task performance (F_1,112_ = 4.17, *p* = 0.097). Regarding the incorrect-loss shift proportion, maternally separated animals initially performed worse at session one, shifting only 54.0 ± 2.7% of the time compared to 63.9 ± 2.0% for controls (t_55.3_ = 3.36, *p* = 0.002), but by session seven there was no difference between groups, with RMS animals shifting 61.2 ± 2.3% of the time and controls shifting 60.9 ± 1.4% of the time (t_55.3_ = −0.10, *p* = 0.92). While post-hoc testing was not performed for overall task performance due to the trend significance level, similar trajectories were evident: RMS females initially touched the correct target only 61.6 ± 2.2% of the time compared to controls at 65.8 ± 0.9 %, but by session seven this difference had closed, with RMS animals scoring 65.5 ± 2.1% and controls scoring 64.7 ± 1.0%.

For males, both response variables had significant group × session interactions. With respect to correct target selection (F_1,172_ = 7.52, *p* = 0.006) and shifting following losses on the incorrect target (F_1,172_ = 9.86, *p* = 0.003), the performance of maternally separated males worsened or remained constant while controls improved over time. Initially, RMS males achieved better task performance, selecting the correct stimulus 68.4 ± 0.9% of the time compared to 65.4 ± 1.0% of the time for controls (t_75.4_ = −2.60, *p* = 0.025), but by day seven this discrepancy had abated, with RMS animals scoring 68.2 ± 0.7% and control animals scoring 69.4 ± 0.6% (t_75.4_ = 0.98, *p* = 0.24). On incorrect-loss shift proportion, RMS animals initially performed better (non-significantly), at 65.9 ± 1.8% shifting vs. 60.5 ± 2.3% shifting for controls (t_91.7_ = −2.25, *p* = 0.063), but by day seven, controls performed significantly better, at 66.5 ± 1.4% shifting vs. 61.3 ± 1.2% shifting for RMS animals (t_91.7_ = 2.16, *p* = 0.007).

The adult stress appeared to have differential effects specifically on the same two metrics that were affected by RMS itself. For overall task performance, there was a significant interaction between group and stress day (F_1,89.4_ = 6.87, *p* = 0.003). Initially, at stress day three, RMS animals performed worse, with 63.1 ± 0.8% correct touches vs. controls with 66.3 ± 0.8% correct (t_106.8_ = 2.58, *p* = 0.006), but performance of RMS animals recovered such that by day eleven there was a trend for worse performance by controls (RMS 67.5 ± 1.2% vs. control 65.2 ± 0.7% correct, t_119.4_ = −1.54, *p* = 0.092). For incorrect-loss shift proportion, there was a trend for a group by stress day interaction that followed the same trajectory as for correct target selection (F_1,89.0_ = 3.26, *p* = 0.058). RMS animals initially performed worse, with 60.1 ± 1.3% shifting compared to 64.4 ± 1.5% shifting, but ultimately had similar shift proportions, with RMS animals at 65.4 ± 2.1% compared to 62.9 ± 1.6% for controls.

### Reward and negative feedback sensitivity explain most of the variability in PRL performance

Across all seven initial PRL sessions, correct-win stay proportion showed only a weak correlation (*ρ* = 0.13) with incorrect-loss shift proportion, indicating that reward sensitivity and negative feedback sensitivity are minimally related and represent distinct aspects of animal behavior, even though negative feedback here is signaled non-reward. Data visualization revealed that both variables had a fully linear relationship with correct touch proportion. In a linear model in which only these two terms were used to predict correct touch proportion, the adjusted R-squared indicated that these metrics together accounted for 81.04% of the variability in task performance. Both correct-win stay proportion (F_1,333_ = 699.56, *p* = 8e-84) and incorrect-loss shift proportion (F_1,333_ = 559.59, *p* = 3e-73) were highly significant. When added to this model, latency to respond was not significant (F_1,332_ = 0.86, *p* = 0.35), indicating that decision latency was unrelated to decision accuracy. Similarly, there was no bivariate correlation between latency to respond and task performance (*ρ* = 0.07).

### Behavioral sequelae of ELS persist beyond any effects on classical ‘depression’ and ‘anxiety’-like behavior

**Fig. 3:**
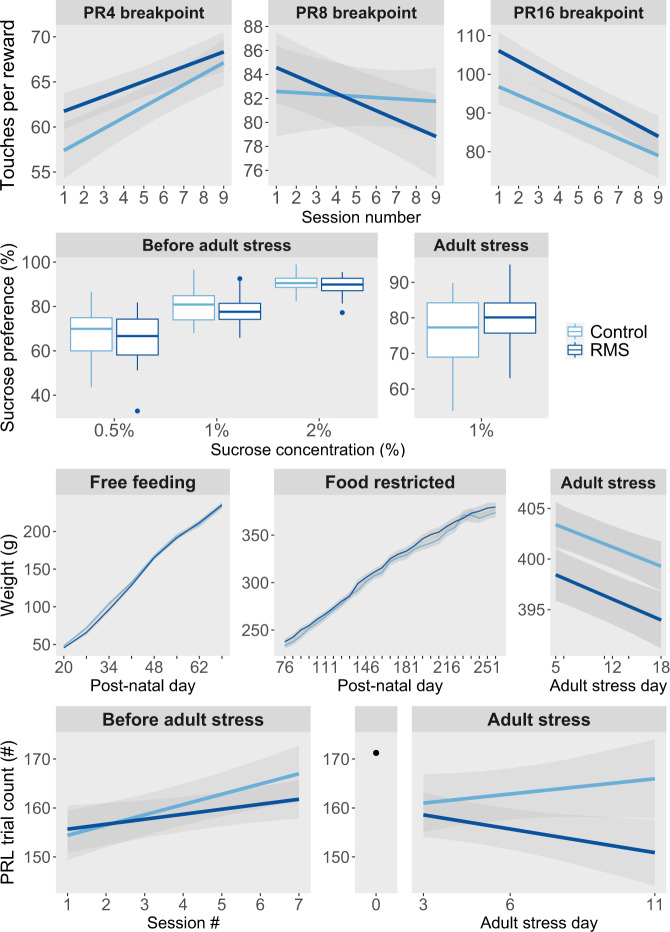
Despite having long-lasting cognitive effects of possible relevance to depression, repeated maternal separation (RMS) did not induce a long-lasting anhedonia. Into adulthood, RMS had no effects on: motivation to work for sucrose reward on three different progressive ratio (PR) schedules of reinforcement (top row), sucrose preference before or during a novel sub-chronic adult stress (upper middle row), body weight before or during the adult stress (lower middle row), and trials completed to earn sucrose reward on the probabilistic reversal learning (PRL) task (lower row).

**Fig. 4:**
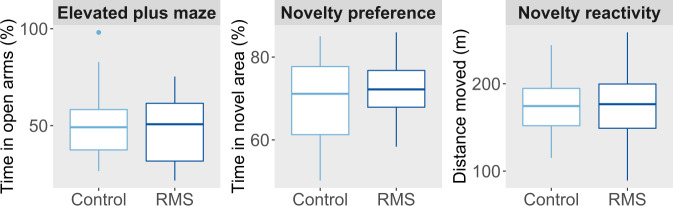
By young adulthood, no effects of repeated maternal separation (RMS) on classical measures of anxiety-like behavior and novelty response were detectable, despite the persistence of several cognitive effects of possible relevance to anxiety. There was no long-lasting effect of RMS on elevated plus maze (EPM) open arm time, time spent on the novel area on the novelty preference test (NPT), and distance moved in the novelty reactivity task (NRT).

Four measurements were collected that can be sensitive to the presence of anhedonia: body weight, sucrose preference, breakpoint on the PR task, and trial count on the PRL task. Additionally, ‘anxiety-like behavior’ was measured using the EPM, and response to novelty was measured using the NPT and NRT. Apart from body weight, all these measures were collected exclusively in adulthood. By this time, both before and during application of a novel sub-chronic stressor, no significant effects of RMS were detectable on any of these measures (Figs. 3, 4). The only trend effect identified was for a main effect of group in the analysis of body weight during adult stress (F_1,50.0_ = 3.61, *p* = 0.062), with stress causing RMS animals to have a slightly lower body weight across the stress period than controls (RMS 396.4 ± 2.4 g vs. controls 401.5 ± 2.1 g).

### ELS is associated with an increase in amygdala volume in the context of adult stress

RMS and control animals underwent MRI scans at three time points: the day following conclusion of RMS (PND 20), in early adulthood (PND 62) in the absence of recent stress, and late adulthood (PND 285) during the application of a chronic adult stressor. Masks of six regions of interest (Figs. S4 and S5) were warped into study template space for each of these time points, and from there into subject space for volume quantitation.

**Fig. 5:**
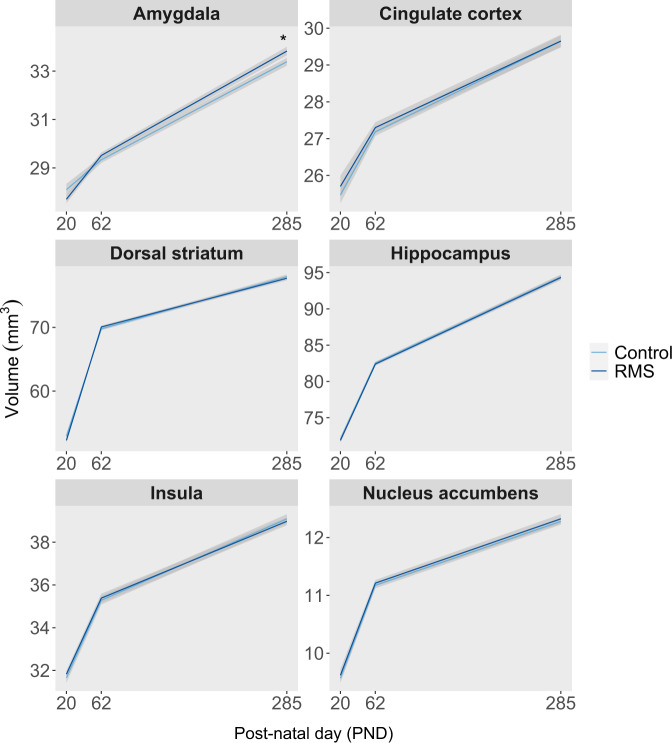
Effects of repeated maternal separation (RMS) on regional brain volumes. Animals underwent MRI scanning on PND 20 (RMS *n* = 17, control *n* = 12), PND 62 (RMS *n* = 22, control *n* = 20), and PND 285 (RMS *n* = 21, control *n* = 23), and data were analyzed to quantify the volumes of six regions of interest. Maternal separation resulted in increased grey matter volume in the amygdala at the timepoint in late adulthood during adult stress, but not in early adulthood or immediately following RMS.

Results are presented in Fig. 5. In the mixed-effects model for amygdala volume, there was a significant group × time point interaction (F_2,66.0_ = 4.72, *p* = 0.018). Post-hoc testing revealed a significant difference at PND 285 (t_91.1_ = −2.20, *p* = 0.044), with RMS animals having an amygdala volume of 33.8 ± 0.178 mm^3^ compared to 33.4 ± 0.161 mm^3^ for controls. There were no differences at PND 62 (t_91.8_ = −0.87, *p* = 0.294), where RMS animals had an amygdala volume of 29.5 ± 0.120 mm^3^ and controls had a volume of 29.3 ± 0.126 mm^3^, or at PND 20 (t_101.2_ = 1.59, *p* = 0.157), where RMS animals had a volume of 27.7 ± 0.162 mm^3^ and controls had a volume of 28.1 ± 0.241 mm^3^. There were no other significant interactions involving group.

## Discussion

The present study investigated whether ELS results in long-lasting functional impairments as measured with a comprehensive behavioral test battery, and whether these coincided with structural abnormalities in selected brain regions of interest. We report a novel, long-lasting phenotype of RMS, based on responding in a probabilistic reversal learning task previously shown to be sensitive to human anxiety and depression [29, 30]. Further, we report that these long-lasting behavioral changes were accompanied by a larger amygdala volume in adulthood when measured during later-life stress.

RMS had long-lasting effects on two distinct aspects of animals’ behavior on the PRL task. First, RMS animals, regardless of sex, were considerably slower to select between the two stimuli on the PRL task. Simultaneously, there was no association between latency to respond and task performance, and instead, over 80% of the variability in task performance was accounted for by inter-individual differences in reward and negative feedback sensitivity. Further, there was no effect of RMS on latency to collect reward, indicating that the latency to respond effect was not a function of differences in locomotor functioning or incentive motivation. This same deficit has been repeatedly reported in human anxiety. Indeed, a recent meta-analysis of 58 studies involving over 8000 subjects concluded that anxiety was associated with slower responding relative to achieved performance, and that this medium to large effect was exacerbated under conditions of higher cognitive load [57]. This effect has even been found on PRL in at least one study, in which subjects with generalized anxiety disorder (GAD) had slower responses on several trial types compared to subjects without GAD, but nevertheless achieved an equivalent number of correct responses [30]. It has been suggested that this finding may result from either premature or delayed disengagement of attention from stimuli, perhaps particularly where a subset of stimuli have a negative valence [58]. Such a deficit could be compensated for by simply spending more time before making a decision, thus allowing accuracy to be preserved at the cost of latency [59]. In addition to its association with anxiety, slowed responding has also been associated directly with ELS, both in humans and non-human primates. In one large study in children aged 6–12, a greater number of caregiver changes was associated with greater slowing of response time by the presence of distractor stimuli on the Flanker task, while simultaneously there was no relationship between caregiver changes and task performance [23]. Additionally, in 4.5–5.5 year old macaques, early-life maltreatment, consisting of rejection and physical abuse by the mother in the first 3 months of life, was associated with globally slower reaction times on a dot-probe task involving a social threat visual stimulus, but not on a nearly-identical task involving no threat-associated stimulus [60]. Our findings add to this literature suggesting that ELS has long-lasting effects on response time in certain decisional contexts. The additional association between delayed responding and anxiety raises the possibilities that some of those deficits may in fact be due to ELS histories in studied subjects, or that these deficits may in some way causally contribute to the development of anxiety.

The other novel and significant behavioral findings reported here relate to reward and negative feedback sensitivity. RMS desensitized females and sensitized males to the negative possible outcomes of their decisions on the task, as indicated by their initially lower and trend-higher incorrect-loss shift proportion respectively. This decreased and increased sensitivity to negative feedback appeared to drive parallel differences in overall task performance, with RMS females initially performing non-significantly worse than controls and RMS males initially performing significantly better. The fact that these initial differences between groups later converged suggests that groups had biased responses to negative outcomes at baseline that were eventually overcome after hundreds of trials. These findings may have some overlap with human anxiety and possibly depression. Regarding depression, while findings from some small, earlier studies suggested that depressed patients may have a higher negative feedback sensitivity on the PRL task [61, 62], the measure used to conclude this (correct-loss shift proportion) would also have been influenced by reward sensitivity, and the findings could hence have been explained by the reduced reward sensitivity that has been repeatedly reported in depression [28, 29, 63–65]. More recent, often larger studies reporting either incorrect-loss shift proportion or all-loss shift proportion have provided evidence that negative feedback sensitivity is either reduced or unaffected in depression [29, 65, 66]. With respect to anxiety, a few studies have been conducted on its relationship to negative outcome and reward sensitivity on the PRL task [30, 65, 67], and one of these is particularly methodologically strong [67]. In this study, among 80 university students, individuals with high trait anxiety had a significantly lower all-loss shift proportion, but with no difference in all-win stay proportion, suggesting that anxiety is associated specifically with reduced negative feedback sensitivity. Altogether, given that RMS resulted in long-lasting sexually dimorphic effects on negative feedback sensitivity on the PRL task, as well as a susceptibility to stress-induced deterioration in task performance, it appears that RMS may cause persistent alterations to cognitive processes relevant at a minimum to human anxiety, and possibly to human depression.

Finally, we reported that while RMS animals had equivalent amygdala volume to controls in young adulthood at age 2 months in the absence of later-life stress, they had increased amygdala volume at age 9.5 months in the presence of a later-life stressor. This implies that RMS interacted either with ongoing neurodevelopment between early and late adulthood or, perhaps more likely, with the presence of later-life stress, to ultimately result in detectable amygdala enlargement. To date, only a small number of ROIs have been examined via MRI for volumetric effects of RMS, specifically: whole hippocampus, dorsal hippocampus, ventral hippocampus, whole cortex, motor cortex, dorsal striatum, and combined infralimbic and prelimbic cortex [68–72]. All measured ROIs have been reported to be unaffected, with the exception of one report of a short-lived effect of decreased hippocampal volume [71]. Thus, prior findings are largely consistent with our hippocampus and dorsal striatum results, while our measurements of other ROIs, including the amygdala, are novel. While further experiments are necessary to identify which interaction was responsible for our amygdala finding, similar results have sometimes been reported in the clinical ELS literature [73–76], particularly in studies not enriched for psychopathology and therefore less burdened statistically by a low signal to noise ratio and confounding [14]. Meanwhile, the preclinical literature is precise in supplying likely mechanisms for this effect, with numerous reports in RMS animals of increased numbers in the amygdala of neurons, dendritic spines, dendritic branches, and presynaptic boutons [42, 77–79]. If enhancements to corticolimbic connectivity, including amygdala connectivity, give way to potentiated responses to further stress, including at the microstructural level, this could explain the apparent interaction [80]. Regarding the anticipated functional consequences of this larger amygdala volume, while amygdala volume has no clear relationship to depression or anxiety [31, 81, 82], a larger volume seems to predict elevated sympathetic nervous stress reactivity and thus immunological stress reactivity [83–87], and, in the context of ongoing stress, blunted HPA stress reactivity [88–90]. Further, given that the amygdala is known to be a core part of the circuit that modulates negative feedback sensitivity in humans [91, 92], it is possible that the effects of RMS on the amygdala played a causal role in mediating the behavioral changes observed on the PRL task.

It is noteworthy that the alterations to RMS animals’ response times and responding to negative outcomes persisted late into adulthood despite the absence of classical “depression-like” or “anxiety-like” behavior, specifically on the SPT and EPM. Our findings on these tasks are broadly consistent with others’ results. RMS does not appear to cause an anhedonia that persists into adulthood, whether on the SPT [93–103] or other tasks [96, 98, 104–106]. These results are also consistent with findings from other rodent chronic stress paradigms, which indicate that anhedonia develops linearly over time with stressor application [107–111], and then recovers linearly within days to weeks following stressor cessation [111–116]. Results on tests of putative anxiety-like behavior are highly inconsistent, with 60% of mouse experiments reporting that maternal separation is anxiogenic on these tests, and 35% reporting that it is anxiolytic [117]. This inconsistency may result from the fact that these tests, while often described as measuring unconditioned anxiety, are in fact highly susceptible to between-group differences in conditioned responding to the experimenter or associated stimuli, which may develop from differential handling or stressor histories [118]. Additionally, these tests, while useful, capture variability only in narrow aspects of behavior, while human disorders such as anxiety and depression have heterogeneous cognitive, behavioral, and emotional manifestations [119–121]. Finally, putting aside the features of the established syndromes, in humans, there are other measurable behavioral and cognitive disturbances that are thought to precede and predispose to depression and anxiety [22, 43]. Our findings add to arguments that measures in rodent studies relating to depression and anxiety causality should extend beyond these conventional tests [122].

## Conclusion

We report novel behavioral and neurobiological effects of ELS as assessed using the repeated maternal separation procedure. RMS interacted with an additional stress in later life or with aging to result in enlargement of the amygdala, a critical component of the corticolimbic circuitry responsible for threat detection and response. RMS also caused long-lasting sexually dimorphic effects on responding to the negative outcome and associated stimuli on the PRL task, and RMS animals were slower to respond on that task, even in the absence of impaired accuracy. We hypothesize that altered corticolimbic processing of negative information, perhaps caused by altered connectivity between the amygdala and other corticolimbic structures, may be responsible for both behavioral effects. Future research in RMS animals should seek to probe the precise cognitive and neurobiological mechanisms for the PRL effects observed, and to examine whether the observed effects may interact with environmental stimuli such as stressors to result in phenotypes with face, construct, etiological, and predictive validity for anxiety or depression.

## Supplementary information

Supplementary Information
