# Supplementary material for: Early-life stress biases responding to negative feedback and increases amygdala volume and vulnerability to later-life stress
Source: Transl Psychiatry. 2023 Mar 7;13:81. doi: 10.1038/s41398-023-02385-7 (PMC9992709; doi:10.1038/s41398-023-02385-7)
Supplement: Supplementary file 1 — Supplementary Information [file 41398_2023_2385_MOESM1_ESM.pdf]

Supplementary Information for:

Early-life stress biases responding to negative feedback and increases amygdala volume and vulnerability to later-life stress

Ethan G. Dutcher [1], Laura Lopez-Cruz [1], E.A. Claudia Pama [1], Mary-Ellen Lynall [2,3], Iris C. R. Bevers [4], Jolyon A. Jones [1], Shahid Khan [5], Stephen J. Sawiak [6,7], Amy L. Milton [1], Menna R. Clatworthy [3], Trevor W. Robbins [1], Edward T. Bullmore [2], Jeffrey W. Dalley [1,2,†]

[1] Department of Psychology, University of Cambridge, Cambridge CB2 3EB, UK; [2] Department of Psychiatry, University of Cambridge, Cambridge CB2 0SZ, UK; [3] Molecular Immunity Unit, MRC Laboratory of Molecular Biology, Cambridge CB2 OQH, UK; [4] Faculty of Medical Sciences, Radboud University, Nijmegen 6525 XZ, The Netherlands; [5] GlaxoSmithKline Research & Development, Stevenage SG1 2NY, UK; [6] Department of Physiology, Development, and Neuroscience, University of Cambridge, Cambridge CB2 3EL, UK; [7] Wolfson Brain Imaging Centre, Department of Clinical Neurosciences, Cambridge CB2 0QQ, UK

Supplementary methods

Experimental timeline

A timeline is provided in Figure S1 that describes the sequence and timing of all procedures that animals underwent. Further detail is provided in subsequent methods sections.

Food restriction

Using an online tool [1], body weight data was extracted from published free-feeding growth curves of male [2] and female [3] Lister-Hooded rats. A linear model was fit for each sex to estimate weight based on animal age. Both models included two terms, one of which was the natural logarithm of animal age, and one of which was an inverse exponential of animal age. R^2 for both models was > 0.99. Model coefficients were then used in an equation that allowed prediction of an individual animal's weight at any age based on a free-feeding measurement collected at any other age. Food restriction involved once-daily administration of an amount of chow titrated daily to maintain each animal above 85% of its individually predicted free-feeding weight.

Adult footshock stress

The number of shocks delivered across the first 8 days for all animals was as follows: 2, 1, 2, 0, 1, 2, 0, 1. The distribution of shock delivery across the remaining 11 days varied between animals but was balanced across group and sex, and animals always received 2 shocks on the day immediately before: each animal's MRI scan day (any of stress days 9-13), the sucrose preference test day (any of stress days 16-17), and the sacrifice day (stress day 19). These varied schedules were necessary to circumvent the limited number of MRI scans possible to conduct on a given day. Animals never received shock sessions on their sacrifice day. The timing of the footshocks within each session was random, except that shocks were never delivered during the first five minutes or last ten minutes of the session. Shock intensity was 0.5 mA and duration was 0.5 seconds. The chambers used were conventional operant chambers (Med Associates, St Albans, VT) equipped only with a grid floor, a house light, high-contrast distinctive wallpaper behind the transparent plexiglass surfaces, and a small overhead camera. A distinctive scent cue was provided by applying 3 drops of eucalyptus globulus essential oil (Neal's Yard Ltd, Cambridge, UK) to a cotton ball and placing it in the sound-attenuating box that enclosed each chamber before each session. The grid floor was connected to a scrambled shock generator (Med Associates).

Arena-based behavioral testing

Three behavioral tasks involved monitoring animal performance in an arena or maze. For each of these tasks, the appropriate plexiglass arena was placed above a 1.1 m × 1.1 m infrared-illuminated platform. Rat movements were recorded from above using an infrared camera (FLIR Systems, Wilsonville, OR, USA), and analyzed using VideoTrack software (ViewPoint Behaviour Technology, Lyon, France). Rats were tested on each of these tasks only once. Testing always occurred during the dark phase of the light cycle, which occurred between 0900 and 2100.

Rats were tested on the elevated plus maze (EPM) between PND 66-69. This task exploits rats' natural fear of open and well-lit places in order to obtain a measure of anxiety [4], calculated as the time spent in the exposed, brightly lit arms as a proportion of the time spent in those arms as well as the closed, darker arms. The maze consisted of a plus-shaped platform with four arms (45 cm length × 10 cm width) elevated 80 cm above the infrared-illuminated base. The closed arms were surrounded by a 45 cm tall opaque wall, while the open arms were surrounded by only a 1 cm tall transparent lip. Rats were placed individually in the center of the maze such that each animal initially faced a closed arm. Illumination was kept at 10-15 lux within the closed arms and 55-65 lux within the open arms. Test time was 5 minutes.

Novelty-induced locomotor reactivity was assessed between PND 70-73. In this task, rats were placed in a 50 cm × 50 cm × 50 cm arena with grey walls and a white floor, and their movements were recorded and tracked. Distance moved across the whole 2-hour session was determined. The arenas were brightly lit at 500-600 lux.

The novelty preference test was performed between PND 74-76. The arena for this task contained two large rectangular chambers (20 cm × 50 cm × 50 cm) divided by a small rectangular alleyway (10 cm × 50 cm × 50 cm). The two large chambers differed in color (white or black) and texture (smooth or dotted with small round pits). Rats were placed for 5 minutes in the central alleyway and allowed to habituate, before a door was opened to one of the other two chambers for 25 minutes. Finally, doors were opened to both chambers for 15 minutes, so that the animal could freely explore all three chambers. Novelty preference was defined as the time spent in the novel chamber (the second available chamber) as a percentage of the combined time spent in the familiar chamber (the first available chamber) and the novel chamber [5]. Illumination within all three chambers was kept very low at < 2 lux.

Sucrose preference test

Sucrose solutions were freshly prepared immediately before provision to animals, using food-grade sucrose (MP Biomedicals, Solon, USA).

At each of the two testing timepoints, animals initially underwent three habituation steps. First, animals were provided with one bottle of 1% sucrose solution in their home cage for 24 hours, in addition to the water normally available via the cage rack water delivery system. After the 24 hours had concluded, animals were placed individually into large cages with clean bedding. One bottle containing 1% sucrose solution and one containing water were then provided. After 45 minutes, the bottles were removed and animals were placed back into their home cages. The next day, each animal was placed into the same cage as before for 45 minutes, but this time the location of the sucrose bottle and water bottle were swapped.

The following day, to conduct the test itself, each animal was again placed into the same cage, but was left there for four hours. The location of each bottle was reversed compared to the previous day, and these locations were again reversed two hours into the test session. At the pre-stress timepoint, tests of preference for the 0.5%, 1%, and 2% sucrose solutions were separated by a 44-hour washout period in the home cage. For a given animal, all sessions (habituation or test) in the test cage commenced at the same time of day, and the daily food ration was always provided approximately 20 hours prior to that time.

The weight of the sucrose and water bottles was weighed before and after each test. For each bottle, the weight after was subtracted from the weight before, giving a measure of absolute sucrose solution and water consumption. Sucrose preference over the four hours was defined as the amount of sucrose solution consumed as a percentage of the total amount of sucrose solution and water consumed.

Operant behavioral testing

Apparatus

Chambers were contained within fan-ventilated, light- and sound-attenuating boxes. One wall of each chamber consisted of a 15-inch LCD touchscreen capable of infrared touch detection (Nexio Co. Ltd, Incheon, Korea), while the opposite wall contained a pellet receptacle with a head-entry detector and accompanying light. Chambers were controlled using the K-Limbic software (Conclusive Marketing Ltd., High Wych, UK).

Progressive ratio schedules of reinforcement

The stimulus was always located in the middle of the screen by width, roughly 2 cm above the metal bar floor. Where a stimulus touch or omission resulted in pellet delivery, a 1 s tone (2.9 kHz) was generated, the magazine light was turned on, and the stimulus was removed from the screen. Upon head entry into the magazine, the magazine light was turned off, and a 5 s inter-trial interval was initiated, after which the stimulus was returned to the screen. Where a stimulus touch did not result in reward delivery, a 20 ms tone was generated and the stimulus was removed from the screen for 0.5 s. The house light remained off at all times. Animals underwent a maximum of one training or test session per day.

Animals progressed through three training stages: (1) for one session only, pellets were delivered even if animals did not touch the stimulus, (2) until animals earned 100 pellets in a given session, a pellet was delivered every time the animal touched the stimulus, and (3) until animals earned 100 pellets in each of 2 sessions, or 60 pellets in each of 5 sessions, a pellet was delivered every time the animal touched the stimulus 5 times. During the initial training session, the stimulus was presented for up to 30 s at a time; if animals touched the stimulus once, or the 30 s period elapsed without any stimulus touch (an omission), reward was delivered as described above. The number of sessions each animal required to progress through the training stages was recorded.

Probabilistic reversal learning

In the first stage of training (touch training A), at the start of each trial, the visual stimulus was randomly presented on either the left or right side of the touchscreen. The second stage of training (touch training B) was identical to the first, except that background touches were punished (with punishment defined in the main text). The third stage of training consisted of a deterministic reversal learning (DRL) task. This task was identical to the PRL task, except that a touch on the correct stimulus resulted in reward on 100% of trials, while a touch on the incorrect stimulus resulted in punishment on 100% of trials.

For each animal, only one chamber session was conducted per day. Animals commenced touch training A approximately 3-4 weeks in advance of the day they were scheduled to commence the adult stress. Animals were progressed to touch training B once they had completed one session of touch training A in which they earned at least 100 rewards. Animals were progressed to the DRL task once they had completed two consecutive sessions of touch training B in which they earned

at least 100 rewards on both occasions. Animals were progressed to the PRL task once they had completed two consecutive sessions of the DRL task in which they achieved at least 4 reversals on both occasions. Once they began touch training, animals completed one session daily of the appropriate task up to and including the first day of the adult stress, when they were tested in the morning and commenced on the stress in the afternoon. After adult stress commencement, they were tested on stress days 3 and 6, and also day 11 where this did not conflict with an MRI scan. Almost all animals completed at least seven sessions of the PRL task before the adult stress began, but many did not complete more sessions than this, so pre-stress analysis was limited to the first seven sessions.

Prior to calculation of the latency means per session, to minimize the impact of extreme intra-session outliers, latency data were winsorized within each session using a conservative threshold of 3.5 median absolute deviations [6–8].

Several measures beyond those discussed in the main text were recorded or calculated. The number of reversals was recorded, as was the average number of perseverations per reversal. Perseverations were defined as serial touches on the newly-incorrect target following reversal, not including the first post-reversal incorrect touch (if one occurred). Thus, if an animal touched the incorrect target twice immediately following reversal, one perseveration would have been scored for that reversal. The incorrect-win stay proportion and correct-loss shift proportion were calculated. For both of the touch training tasks and for the DRL task, the number of sessions each animal took to achieve criterion and thus progress to the next task was recorded.

MRI

MRI scanning

After induction using 3-5% isoflurane, 1-2% isoflurane was delivered in 100% oxygen at 1 L/min. During the scan, rats were monitored using a pulse oximeter, respiratory tracer pad, and rectal temperature probe (SA Instruments, Stony Brook, NY, USA). Vital signs (heart rate, respiratory rate, oxygen saturation, body temperature) were maintained within strain-specific norms via adjustment of anesthesia depth and the temperature of a heat pad secured atop the animal.

Image acquisition

A three-dimensional gradient echo sequence was used to acquire structural images. The field of view of 25.60 × 20.48 × 30.72 mm^3 was constrained within a matrix of 160 × 128 × 192 voxels, each with an isotropic resolution of 160 μm^3. Three different image types were obtained, with each providing unique tissue contrast: magnetization transfer (MT) images, proton density (PD) images, and T1-weighted images. PD images were acquired using a relaxation time (TR) of 25 ms and an echo time (TE) of 2.41 ms, with a flip angle (FA) of 6°. MT images were acquired using the same parameters, but with the additional application of 4 ms radiofrequency (RF) pulses with a Gaussian shape (2 kHz frequency offset, bandwidth 685 Hz, 10 uT magnitude). T1 images were acquired using a TR of 18 ms, a TE of 2.41 ms, and an FA of 40°. Acquisition was accelerated by a factor of 1.55. Images were acquired every 2.1 ms until 6 MT, 6 T1, and 8 PD images were acquired, and then for each image type, a single average image was produced.

Image pre-processing

All MRI processing operations were performed using tools included in: the Advanced Normalization Tools (ANTs) toolbox v2.3.4 [9,10], FMRIB Software Library (FSL) v6.0 [11,12], MRTrix3 v3.0.4 [13], and convert3d v1.1.0 [14].

First, bias field correction was performed using *N4BiasFieldCorrection* (ANTs) [15] on MT, T1, and PD images. Depending on the image type, between 6-18 bias-corrected images were generated for each input image, each using a different b-spline mesh, and then corrected images were averaged without normalization to produce the final bias-corrected image.

Because MT, T1, and PD images from the same animal at a given timepoint were not perfectly aligned with each other, the rigid transformations from bias-corrected T1 and PD to MT space was found for each set of those three images using *antsRegistration*. This transformation was then converted to MRTrix3 format using *c3d_affine_tool* (convert3d) and *transformconvert* (MRTrix3), and then applied to the header of the bias-corrected T1 or PD image using *mrtransform* (MRTrix3) to avoid resampling.

Generation of study templates

Tri-modal template generation was performed using *antsMultivariateTemplateConstruction2* (ANTs). At each timepoint (PND 20, 62, and 285), one animal's scan was selected to act as the initial reference space, and then sequentially for all other scanned animals, the three bias-corrected

images (MT, T1, PD) were concurrently rigidly registered into the reference space. The transformed images were averaged with normalization to produce three modality- and study-specific template images (referred to as "study templates"), and then each animal's original bias-corrected images were non-linearly registered to the new templates. This average-and-register step was performed a total of four times in sequence: twice with a three-stage symmetric normalization (SyN) registration, and then twice with a four-stage SyN registration involving 30-50 iterations at the final stage [16]. In all multi-modal registrations, the MT, T1, and PD images were given relative weights of 2:1:1 in calculation of the image metric, due to the superior grey-white matter contrast in the MT images.

Extensive optimization of registration parameters, including whether and where to use brain masks, was performed. Ultimately, regarding masking and brain extraction, the following methods were used: For the PND 20 timepoint only, the template brain was manually masked, and the mask then inverse warped into subject space. Each subject brain mask was manually edited for accuracy, and then the entire template generation process was repeated using brain-extracted images. For the PND 62 and 285 timepoints, template generation was performed using whole-head images without supplying *antsRegistration* with any masks. A final, additional registration to the template was then calculated for use in analyses. This registration used whole-head images, but the affine part was performed by supplying *antsRegistration* a mask of the fixed image only, while the non-linear part used no masks.

ROI volume quantitation

Region of interest (ROI) masks were derived from the Tohoku University Rat Brain Atlas, which was kindly provided by Dr Akira Sumiyoshi of the National Institutes for Quantum and Radiological Science and Technology, Japan (personal correspondence, January 2021). The creation of this atlas is described in Liang et al. [17] and Valdés-Hernández et al. [18]. The current atlas as received from Dr Sumiyoshi was a composite of 46 × 2 unilateral cortical ROIs from the original Tohoku University cortex-only atlas [18], together with 26 × 2 unilateral subcortical ROIs derived from the Calabrese et al. atlas [19], and 3 × 2 unilateral cortical ROIs derived from the Schwarz et al. atlas [20]. The Valdés-Hernández et al. [18] and Calabrese et al. [19] segmentations were based on Paxinos and Watson 6e [21] while the Schwarz [20] segmentations were based on Paxinos and Watson 4e [22].

First, all ROI masks were extracted from the atlas. Then, after unilateral masks were merged to form bilateral masks, the Cg1 and Cg2 masks dividing the cingulate cortex were merged, and the six masks dividing the insular cortex were merged. Non-linear transformations were identified from the Tohoku template image to the PND 285 MT study template image, and from the PND 285 template to the PND 20 and PND 62 templates. These transformations were used to warp select ROI masks into each of the three study template spaces. A mask of grey and white matter together was created for each study template, based initially on the range of voxel intensity values across these two tissue classes, then with further manual editing for accuracy. These intensity masks were applied to the ROI masks to remove any aberrant voxels in CSF, meningeal tissue, or air spaces. ROI visualizations were produced using MRIcroGL [23].

Six ROIs were selected for volume quantitation, including four subcortical ROIs (amygdala, nucleus accumbens, dorsal striatum, and hippocampal formation), and two cortical ROIs (cingulate cortex and insula). The amygdala mask encompasses all amygdaloid nuclei delineated in Paxinos and Watson [21], including the basolateral, central, medial, intercalated, lateral, posterolateral cortical, and posteromedial cortical amygdaloid nuclei [24]. The nucleus accumbens (NAc) mask encompasses both the core and the shell. Sub-segmentation of ROIs was not attempted because almost all of their internal boundaries are not visible using any of the three contrasts acquired here. Non-linear registration depends on intensity boundaries or gradients and cannot attend to invisible boundaries, and thus volume quantitation of sub-regions would be unreliable due to the considerable or predominant influence of the affine part of the registration on the size of the final, subject-space ROI mask.

ROI masks were inverse-warped from each study template space back to subject space. The gray-white matter mask for each template space was also inverse-warped to subject space, to provide an index of total brain volume (TBV). All warping of ROI masks from the PND 285 template space onward was performed using linear interpolation, and ultimately ROI volumes were calculated by multiplying the mean intensity value (range $1 \geq x > 0$) of non-zero voxels by the total volume (in mm^3) occupied by non-zero voxels.

Blinding

Experimenters were not explicitly blinded to experimental condition.

Data analysis

Visualizations were produced using the packages *ggplot2* [25], *corrplot* [26], and *patchwork* [27].

Categorical predictors were handled using sum-to-zero coding. Where mixed-effects models were used: model fitting used restricted maximum likelihood estimation [28], model fitting was performed using the *lme4* package, v1.1-29 [28], models included a random intercept for each subject with no further random effects, and degrees of freedom were approximated using the Kenward-Roger method [29–31].

After model fitting, model assumptions were checked using diagnostic plots. Normality of residuals was evaluated using quantile-quantile plots while homogeneity of variance and linearity were evaluated using plots of residuals vs fitted values. For linear mixed-effects models, diagnostic plots were generated using the *redres* package v0.0.0.9 [32], whereas for logistic mixed-effects models, diagnostic plots were generated using the *dharma* package v0.4.3 [33], which also confirmed no over- or under-dispersion of the model residuals. A data point was considered to be influential, i.e. to unduly influence the model, when it had a Cook's distance of ≥ 1. When any assumption appeared to be violated, or there was at least one influential point, nonparametric methods were used: permutation testing was used for hypothesis testing, and nonparametric bootstrapping was used for descriptive statistics [34–36].

Type II F-statistics were calculated from mixed-effects models using the *KRmodcomp* function in the *pbkrtest* package v0.5.1 [29], and from fixed-effects models using the base R *anova* function. For post-hoc comparisons, two-sample t- (parametric) or z- (permutation test) statistics were calculated using the *emmeans* or *emtrends* functions from the package *emmeans* v1.7.3 [31]. No multiple comparisons correction was performed.

For permutation testing, the Freedman-Lane method was used [37]. Specifically, for each predictor, a larger model was fitted that included the predictor but no higher-order terms, and a reduced model was fitted that excluded the predictor. An F-statistic was calculated which compared these models. Then, to generate a reference null distribution for this F-statistic, 10,000 permutations (complete, random samples without replacement) of the animal identifier were generated using the *sample* function in base R, in a data table consisting of the mapping from animal identifier to any between-subjects variables (e.g. group and sex). This permuted mapping was then merged with a data table consisting of the residuals from the reduced model and the corresponding animal

identifier. These residuals retained the random but not fixed effects from the reduced model. The fuller model and reduced model were then both fitted to each permuted dataset, and compared via calculation of an F-statistic, resulting in a null distribution of 10,000 F-statistics. Finally, the p-value was calculated as the proportion of permutation-derived F-statistics that were equal to or larger than the F-statistic derived from the unpermuted data. For post-hoc comparisons, an identical procedure was followed, using a larger model that included the interaction term to be followed up, and a reduced model that excluded it, except that for a given post-hoc comparison, a z-statistic was calculated from the larger model, and compared to a null distribution of z-statistics derived from a set of 10,000 larger models fit to permuted datasets.

Estimated marginal means (EMMs) were calculated using the *emmeans* R package [31], and except in the case of nonparametric bootstrapping, standard errors were also derived using the same package. To obtain EMMs and associated standard errors via nonparametric bootstrapping, animal identifiers were first resampled (using *sample* in base R) within each group-sex condition 10,000 times with replacement, using original within-condition sample sizes. All outcome and predictor data corresponding to each resampled animal identifier were then pulled from the original data to create a resampled dataset, which the relevant model was then fit to. From each model, any appropriate EMMs were calculated. To obtain the final estimate of a given EMM, the mean of the 10,000 sample-derived EMMs was calculated, and the standard error was calculated by determining the quantiles corresponding to the probabilities 0.1587 and 0.8413 (z-score -1 and +1), after slight expansion of these probabilities to adjust for the moderately small sample sizes [38–40], and dividing the difference in these quantiles by two.

Box plots were produced from appropriate partial residuals, which were calculated using *ggemmeans* from the *ggeffects* package v1.1.4.

Observations were only excluded from analysis in case of documented animal illness.

Code availability: analysis scripts are available upon request.

Body weight analysis

For analysis of pre-stress body weight, age was divided into bins of seven days each, starting from PND 20, and each animal's weight measurements were averaged for each bin. Because animals commenced adult stress at different ages, weights taken during adult stress were excluded from

calculation of per-animal averages, and analysis extended only to the bin starting at PND 258, because large numbers of animals progressively commenced the adulthood stress shortly after that age. Because weight was not linear over time, age was treated as a categorical variable. For post-stress analysis, no binning was performed, and stress day was treated as a continuous variable because animals' weights were linear over time. Post-stress analyses were adjusted for mean-centered baseline, which consisted of animals' median weight over the thirteen days before the first stress day.

Behavioral data analysis

Model structures are reported in the statistics tables in the supplementary information. For adult stress analyses, models included baseline as a predictor [41–43]. For PRL analyses, a baseline for each metric was created for each animal by taking the median over its final five pre-stress PRL sessions. For SPT analysis, each animal's total pre-stress sucrose preference was used as its baseline, defined as the total sucrose solution consumed over the three pre-stress tests as a percentage of the total amount of sucrose and water consumed in that time. Before inclusion in models, baseline variables were first mean-centered.

Body weight was measured at varying intervals from PND 20 through sacrifice. For analysis of pre-stress body weight, age was divided into bins of seven days each, starting from PND 20, and each animal's weight measurements were averaged for each bin. Because animals commenced adult stress at different ages, weights taken during adult stress were excluded from calculation of per-animal averages, and analysis extended only to the bin starting at PND 258, because large numbers of animals progressively commenced the adulthood stress shortly after that age. Because weight was not linear over time, age was treated as a categorical variable. For post-stress analysis, no binning was performed, and stress day was treated as a continuous variable because animals' weights were linear over time.

For PR and PRL analyses, all models treated session number as a continuous variable, to minimize type II error by conserving degrees of freedom and because no frankly non-linear relationships between session number and any response variables were apparent on exploratory data visualization. Where a two-term interaction involving group and session was significant with no significant higher-order interactions, this was followed up with a pairwise comparison between

groups at both the first and last session. Where a three-term interaction involving group, sex, and session was significant, a new model was fit for each sex.

MRI data analysis

A separate linear mixed-effects model was constructed for each ROI, which included the full interaction of group, sex, and timepoint, as well as TBV and its interaction with timepoint. As there were only three timepoints and ROI volume was never linear across them, timepoint was treated as a categorical variable. TBV was centered around the mean TBV at each timepoint. For some ROIs, model residuals were modestly non-normal; for consistency, non-parametric statistics were calculated and presented in the main text for all models, but parametric statistics are similar and are also presented in the supplementary information.

Supplementary figures

Procedure or test	Start range (PND)	End range (PND)
Repeated maternal separation	5	19
1st blood collection & 1st MRI scan	20	N/A
2nd blood collection	53-55	N/A
2nd MRI scan	61-62	N/A
Elevated plus maze	66-69	N/A
Novelty reactivity test	70-73	N/A
Novelty preference test	74-76	N/A
Progressive ratio task (including training)	80-84	115-150
Sucrose preference test (before adult stress)	143-161	147-165
Probabilistic reversal learning (including training)	241-277	stress day 6 or 11
Adult stress start	260-304;<br>stress day 0	N/A
3rd MRI scan	271-309;<br>stress days 9-13	N/A
Sucrose preference test (during adult stress)	276-321;<br>stress days 16-17	N/A
Sacrifice (inc. 3rd blood collection)	279-323;<br>stress day 19	N/A

Figure S1. Experimental timeline. The range of post-natal days (PND) for the start and end of each procedure underwent by repeated maternal separation (RMS) and control animals are provided.

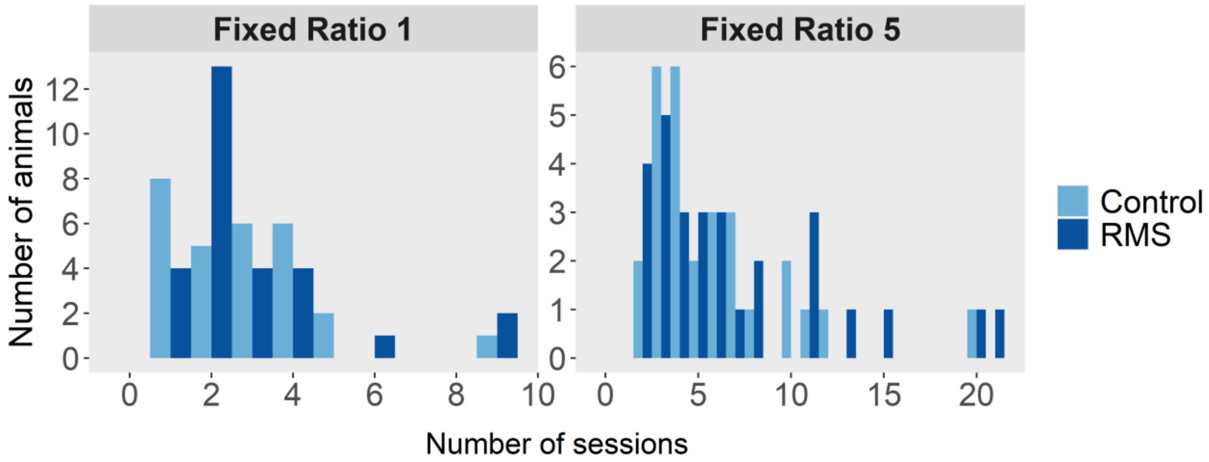

Figure S2. Repeated maternal separation (RMS) did not affect sessions-to-criterion for either of the training stages for the progressive ratio (PR) task. Before being tested on the three progressive ratio (PR) schedules of reinforcement, animals were trained on fixed ratio 1 and 5 schedules, in which they had to respond on the target one and five times respectively to earn a reward. There were no differences in sessions-to-criterion between RMS (n = 28) and control (n = 28) animals. Histogram bins are one session in width.

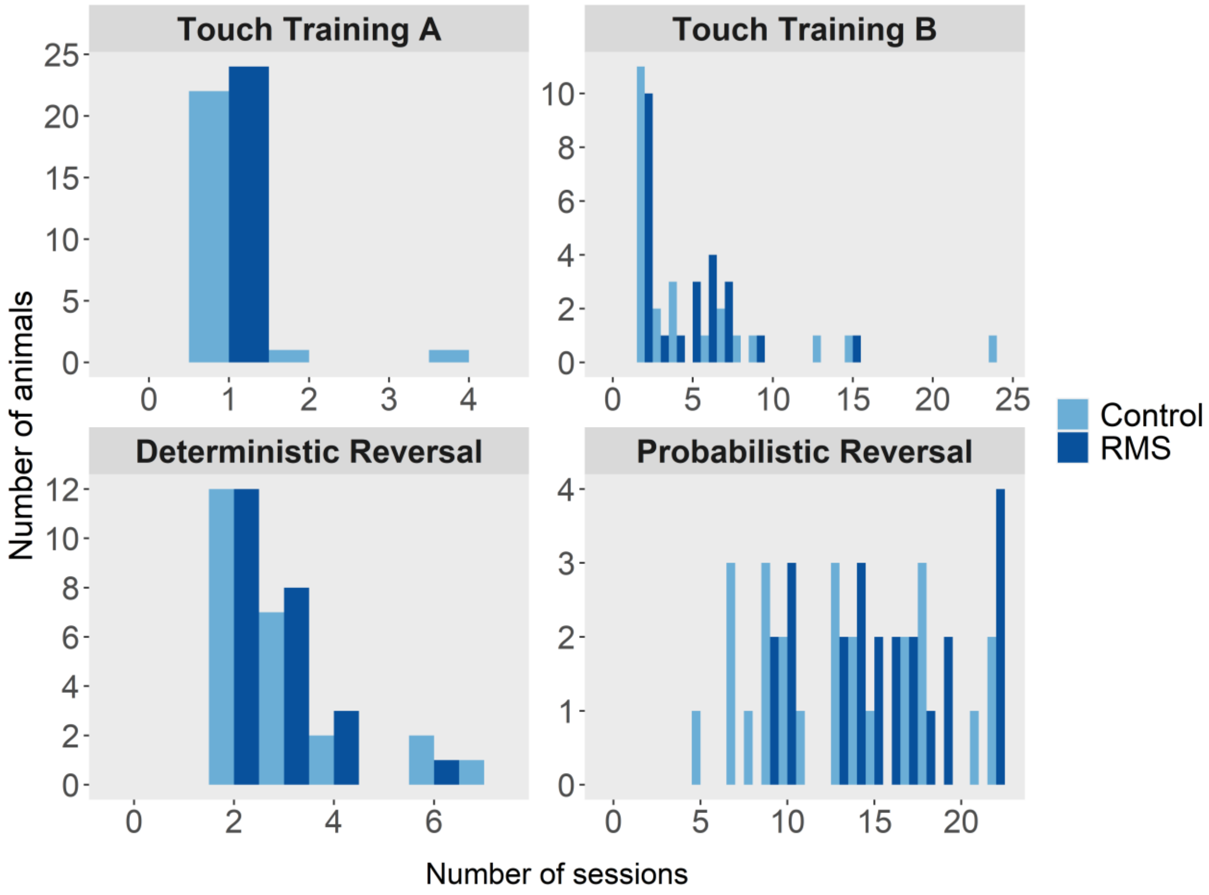

Figure S3. Repeated maternal separation (RMS) did not affect sessions-to-criterion on any training stages for probabilistic reversal learning (PRL) or PRL session count before adult stress. Among animals included in the pre-adult stress analysis, there were no significant differences between repeated maternal separation (RMS, n = 24) and control (n = 24) animals in sessions-to-criterion on the touch training A, touch training B, or deterministic reversal learning (DRL) training stages. Animals included in the post-stress analysis completed varying numbers of PRL sessions before being started on the adult stress (lower right panel; MS n = 25, control n = 23). Histogram bins are one session in width.

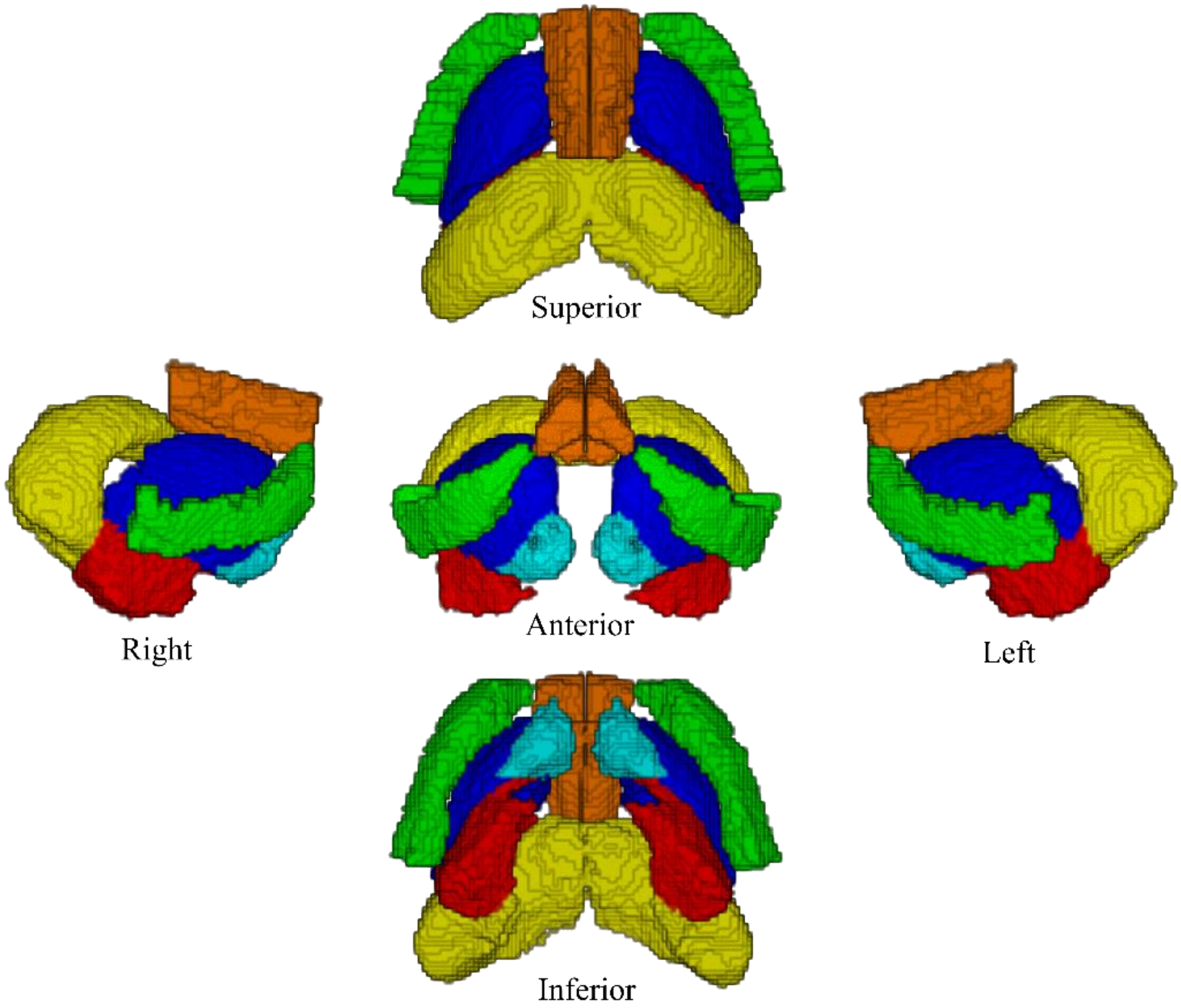

Figure S4. Three-dimensional renderings of region of interest (ROI) masks in PND 285 study template space. ROI masks were obtained by registering the Tohoku University Rat Brain Atlas template image to the PND 285 study template image, and then warping the Tohoku masks into study template space. The six ROIs depicted are: insula (green), cingulate cortex (orange), dorsal striatum (dark blue), ventral striatum (light blue), amygdala (red), and hippocampus (yellow).

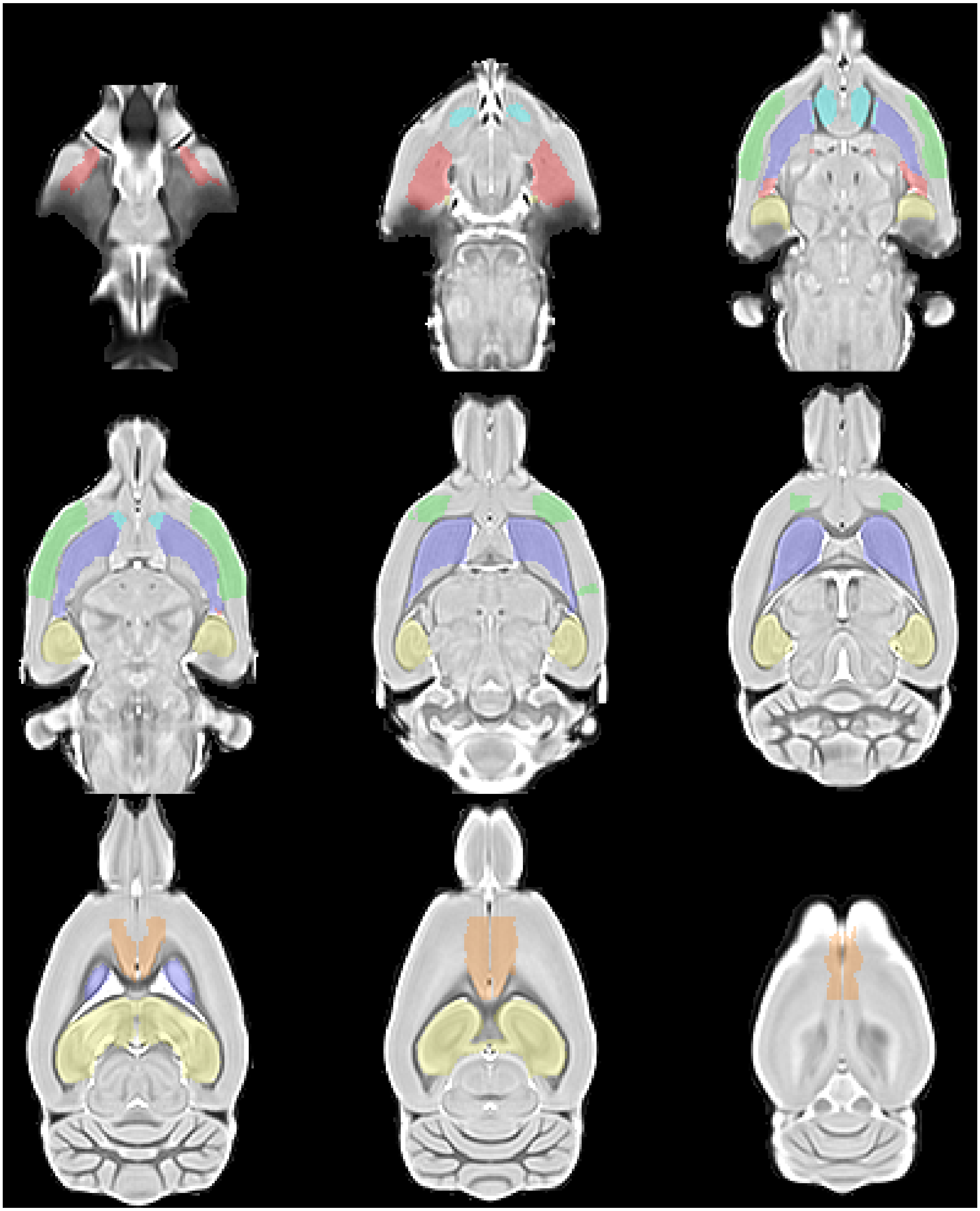

Figure S5. Axial sections of the post-natal day (PND) 285 magnetization transfer (MT) study template, overlaid with the region of interest (ROI) masks. The six ROIs depicted are: insula (green), cingulate cortex (orange), dorsal striatum (dark blue), ventral striatum (light blue), amygdala (red), and hippocampus (yellow).

Outcome	Predictor	F	Df	p (par.)	p (per.)	EMM order	EMMs (bootstrap)	EMMs (parametric)
Correct-loss shift % (PRL BAS)	Group	0.21	1,45.0	0.65	0.65	Control; MS	59.4 ± 1.4; 58.3 ± 1.6	59.4 ± 1.5; 58.3 ± 1.5
	Sex	2.31	1,45.0	0.14	0.14	Female; Male	57.3 ± 1.6; 60.4 ± 1.3	57.3 ± 1.6; 60.5 ± 1.3
	G×S	0.17	1,44.0	0.68	0.68			
	Group×Sess.	1.48	1,285.0	0.23	0.21			
	Sex×Sess.	1.54	1,285.0	0.22	0.21			
	G×S×Sess.	0.88	1,284.0	0.35	0.33			
	Session	2.09	1,287.0	0.15				
Correct-win stay % (PRL BAS)	Group	0.33	1,45.0	0.57	0.58	Control; MS	81.3 ± 0.8; 82.0 ± 1.4	81.2 ± 1.1; 82.0 ± 1.1
	Sex	15.96	1,45.0	2e-04	3e-04	Female; Male	78.6 ± 1.5; 84.6 ± 0.8	78.6 ± 1.2; 84.6 ± 1.0
	G×S	0.14	1,44.0	0.71	0.71			
	Group×Sess.	0.00	1,285.0	1.00	1.00			
	Sex×Sess.	2.00	1,285.0	0.16	0.15			
	G×S×Sess.	0.63	1,284.0	0.43	0.42			
	Session	5.88	1,287.0	0.016				
Incorrect-loss shift % (PRL BAS)	Group	1.79	1,45.0	0.19	0.19	Control; MS	63.0 ± 0.9; 60.6 ± 1.1	63.0 ± 1; 60.6 ± 1
	Sex	6.18	1,45.0	0.017	0.015	Female; Male	60.0 ± 1.1; 63.6 ± 0.9	60.0 ± 1.1; 63.6 ± 0.9
	G×S	3.20	1,44.0	0.081	0.077			
	Group×Sess.	0.83	1,285.0	0.36	0.39			
	Sex×Sess.	0.19	1,285.0	0.66	0.68			
	G×S×Sess.	15.24	1,284.0	1e-04	1e-04			
	Session	0.61	1,287.0	0.44				
Incorrect-win stay % (PRL BAS)	Group	0.47	1,45.0	0.50	0.50	Control; MS	70.6 ± 1.9; 72.6 ± 1.6	70.5 ± 1.8; 72.6 ± 1.8
	Sex	11.74	1,45.0	0.001	0.002	Female; Male	67.3 ± 1.8; 75.9 ± 1.7	67.3 ± 2.0; 75.8 ± 1.6
	G×S	0.43	1,44.0	0.52	0.52			
	Group×Sess.	0.09	1,285.0	0.77	0.82			
	Sex×Sess.	1.71	1,285.0	0.19	0.30			
	G×S×Sess.	2.24	1,284.0	0.14	0.23			
	Session	2.82	1,287.0	0.094				
Latency to collect (s) (PRL BAS)	Group	0.65	1,45.0	0.42	0.42	Control; MS	1.19 ± 0.05; 1.25 ± 0.04	1.19 ± 0.04; 1.25 ± 0.04
	Sex	0.35	1,45.0	0.56	0.56	Female; Male	1.20 ± 0.05; 1.24 ± 0.04	1.20 ± 0.05; 1.24 ± 0.04
	G×S	0.38	1,44.0	0.54	0.54			
	Group×Sess.	1.12	1,285.0	0.29	0.34			
	Sex×Sess.	1.85	1,285.0	0.17	0.21			
	G×S×Sess.	2.29	1,284.0	0.13	0.15			
	Session	0.00	1,287.0	0.97				

Latency to respond (s) (PRL BAS)	Group	10.00	1,45.0	0.003	0.003	Control; MS	1.20 ± 0.06; 1.66 ± 0.11	1.20 ± 0.1; 1.65 ± 0.1
	Sex	0.07	1,45.0	0.80	0.80	Female; Male	1.41 ± 0.08; 1.44 ± 0.10	1.41 ± 0.11; 1.44 ± 0.09
	G×S	1.04	1,44.0	0.31	0.31			
	Group×Sess.	0.18	1,285.0	0.67	0.73			
	Sex×Sess.	0.02	1,285.0	0.90	0.92			
	G×S×Sess.	0.26	1,284.0	0.61	0.69			
	Session	21.01	1,287.0	7e-06				
Perseverations per reversal (PRL BAS)	Group	0.14	1,45.0	0.71	0.71	Control; MS	0.883 ± 0.071; 0.966 ± 0.116	0.883 ± 0.086; 0.967 ± 0.088
	Sex	0.80	1,45.2	0.37	0.38	Female; Male	0.982 ± 0.121; 0.867 ± 0.066	0.983 ± 0.096; 0.867 ± 0.077
	G×S	1.96	1,44.2	0.17	0.17			
	Group×Sess.	1.32	1,281.7	0.25	0.16			
	Sex×Sess.	0.00	1,281.9	0.97	0.97			
	G×S×Sess.	0.45	1,280.9	0.50	0.39			
	Session	0.48	1,283.7	0.49				
Correct touch % (PRL BAS)	Group	0.01	1,45.0	0.91	0.90	Control; MS	66.3 ± 0.5; 65.9 ± 0.8	66.3 ± 0.6; 65.9 ± 0.6
	Sex	16.41	1,45.0	2e-04	1e-04	Female; Male	64.4 ± 0.8; 67.8 ± 0.4	64.4 ± 0.6; 67.8 ± 0.5
	G×S	2.50	1,44.0	0.12	0.12			
	Group×Sess.	0.18	1,285.0	0.67	0.71			
	Sex×Sess.	0.19	1,285.0	0.66	0.70			
	G×S×Sess.	11.28	1,284.0	9e-04	0.001			
	Session	5.47	1,287.0	0.020				
Reversal count (PRL BAS)	Group	0.33	1,45.0	0.57	0.57	Control; MS	3.71 ± 0.16; 3.84 ± 0.23	3.71 ± 0.20; 3.84 ± 0.21
	Sex	17.64	1,45.0	1e-04	0	Female; Male	3.17 ± 0.22; 4.39 ± 0.19	3.16 ± 0.23; 4.38 ± 0.18
	G×S	0.34	1,44.0	0.56	0.56			
	Group×Sess.	0.76	1,285.0	0.38	0.42			
	Sex×Sess.	0.76	1,285.0	0.39	0.41			
	G×S×Sess.	0.02	1,284.0	0.89	0.90			
	Session	7.87	1,287.0	0.005				
Trial count (PRL BAS)	Group	0.13	1,45.0	0.72	0.72	Control; MS	160.7 ± 4.2; 158.7 ± 3.6	160.7 ± 4.0; 158.7 ± 4.1
	Sex	15.59	1,45.0	3e-04	4e-04	Female; Male	148.6 ± 4.2; 170.8 ± 3.6	148.6 ± 4.4; 170.8 ± 3.6
	G×S	0.00	1,44.0	0.97	0.97			
	Group×Sess.	1.94	1,285.0	0.16	0.25			

	Sex×Sess.	0.64	1,285.0	0.42	0.51			
	G×S×Sess.	3.31	1,284.0	0.070	0.13			
	Session	7.15	1,287.0	0.008				
Incorrect-loss shift % [M] (PRL BAS)	Group	0.00	1,27.0	0.95	0.95	Control; MS	63.5 ± 1.3; 63.6 ± 1.2	63.5 ± 1.2; 63.6 ± 1.2
	Group×Sess.	9.86	1,172.0	0.002	0.003			
	Session	0.09	1,173.0	0.76				
Correct touch % [M] (PRL BAS)	Group	1.14	1,27.0	0.29	0.30	Control; MS	67.4 ± 0.6; 68.3 ± 0.6	67.4 ± 0.6; 68.3 ± 0.6
	Group×Sess.	7.52	1,172.0	0.007	0.006			
	Session	5.43	1,173.0	0.021				
Incorrect-loss shift % [F] (PRL BAS)	Group	5.08	1,17.0	0.038	0.038	Control; MS	62.4 ± 1.3; 57.6 ± 1.9	62.4 ± 1.5; 57.6 ± 1.5
	Group×Sess.	6.25	1,112.0	0.014	0.016			
	Session	0.77	1,113.0	0.38				
Correct touch % [F] (PRL BAS)	Group	1.16	1,17.0	0.30	0.30	Control; MS	65.2 ± 0.8; 63.6 ± 1.5	65.2 ± 1.1; 63.5 ± 1.1
	Group×Sess.	4.17	1,112.0	0.044	0.097			
	Session	0.99	1,113.0	0.32				
Correct-loss shift % (PRL DAS)	Group	0.10	1,42.2	0.75	0.75	Control; MS	59.8 ± 1.4; 61.1 ± 2.0	59.9 ± 1.7; 61.0 ± 1.8
	Sex	1.93	1,41.7	0.17	0.17	Female; Male	58.8 ± 1.8; 62.1 ± 1.7	58.8 ± 1.9; 62.1 ± 1.6
	Baseline	2.74	1,41.2	0.11	0.10			
	G×S	1.46	1,41.5	0.23	0.24			
	Group×SD	2.70	1,86.9	0.10	0.12			
	Sex×SD	0.26	1,86.6	0.61	0.62			
	G×S×SD	0.41	1,85.6	0.52	0.53			
	Stress day	3.35	1,88.9	0.070				
Correct-win stay % (PRL DAS)	Group	0.14	1,43.4	0.71	0.72	Control; MS	80.8 ± 0.9; 80.3 ± 1.0	80.9 ± 0.9; 80.3 ± 0.9
	Sex	0.01	1,43.9	0.92	0.92	Female; Male	80.4 ± 1.1; 80.7 ± 1.1	80.5 ± 1.0; 80.7 ± 0.9
	Baseline	25.63	1,45.7	7e-06	0			
	G×S	0.09	1,42.6	0.76	0.76			
	Group×SD	0.22	1,86.6	0.64	0.57			
	Sex×SD	0.04	1,86.2	0.83	0.79			
	G×S×SD	0.44	1,84.8	0.51	0.42			
	Stress day	0.01	1,88.6	0.91				
Incorrect-loss shift % (PRL DAS)	Group	0.96	1,42.5	0.33	0.33	Control; MS	63.8 ± 1.0; 62.2 ± 1.1	63.8 ± 1.1; 62.3 ± 1.1
	Sex	6.70	1,42.3	0.013	0.016	Female; Male	61.0 ± 1.1; 65.1 ± 1.1	61.0 ± 1.2; 65.1 ± 1.0
	Baseline	3.82	1,44.9	0.057	0.060			
	G×S	0.00	1,40.9	0.97	0.97			
	Group×SD	3.26	1,89.0	0.075	0.058			
	Sex×SD	0.03	1,88.2	0.87	0.86			
	G×S×SD	0.37	1,87.4	0.54	0.51			
	Stress day	0.93	1,90.9	0.34				

Incorrect-win stay % (PRL DAS)	Group	1.54	1,43.3	0.22	0.22	Control; MS	76.4 ± 1.9; 72.8 ± 2.4	76.3 ± 2.2; 72.8 ± 2.3
	Sex	0.09	1,43.2	0.77	0.78	Female; Male	74.2 ± 2.4; 75.0 ± 2.4	74.2 ± 2.5; 74.9 ± 2.1
	Baseline	5.04	1,44.1	0.030	0.031			
	G×S	0.45	1,42.6	0.51	0.51			
	Group×SD	2.15	1,86.1	0.15	0.17			
	Sex×SD	1.21	1,85.7	0.27	0.30			
	G×S×SD	0.32	1,84.6	0.57	0.60			
	Stress day	0.87	1,88.1	0.35				
Latency to collect (s) (PRL DAS)	Group	1.88	1,43.5	0.18	0.18	Control; MS	1.28 ± 0.03; 1.32 ± 0.02	1.28 ± 0.02; 1.32 ± 0.03
	Sex	6.66	1,43.2	0.013	0.012	Female; Male	1.26 ± 0.02; 1.35 ± 0.02	1.26 ± 0.03; 1.35 ± 0.02
	Baseline	150.62	1,41.1	2e-15	0			
	G×S	0.89	1,41.9	0.35	0.35			
	Group×SD	1.64	1,83.8	0.20	0.19			
	Sex×SD	0.49	1,83.7	0.49	0.47			
	G×S×SD	0.01	1,82.6	0.94	0.93			
	Stress day	0.06	1,85.9	0.81				
Latency to respond (s) (PRL DAS)	Group	8.94	1,42.9	0.005	0.003	Control; MS	1.29 ± 0.08; 1.69 ± 0.12	1.29 ± 0.10; 1.69 ± 0.11
	Sex	3.13	1,42.5	0.084	0.086	Female; Male	1.36 ± 0.11; 1.61 ± 0.10	1.36 ± 0.11; 1.62 ± 0.10
	Baseline	23.12	1,40.1	2e-05	0			
	G×S	0.59	1,41.3	0.45	0.44			
	Group×SD	0.00	1,87.1	0.96	0.96			
	Sex×SD	0.29	1,86.7	0.59	0.58			
	G×S×SD	1.61	1,85.5	0.21	0.19			
	Stress day	0.06	1,89.2	0.81				
Perseverations per reversal (PRL DAS)	Group	0.68	1,41.1	0.41	0.41	Control; MS	0.840 ± 0.113; 0.673 ± 0.104	0.843 ± 0.100; 0.665 ± 0.110
	Sex	8.72	1,40.1	0.005	0.005	Female; Male	0.960 ± 0.144; 0.553 ± 0.065	0.958 ± 0.111; 0.551 ± 0.093
	Baseline	1.30	1,39.7	0.26	0.26			
	G×S	1.74	1,38.6	0.20	0.19			
	Group×SD	0.63	1,84.4	0.43	0.41			
	Sex×SD	1.18	1,84.1	0.28	0.25			
	G×S×SD	0.91	1,82.8	0.34	0.31			
	Stress day	0.00	1,86.9	0.98				
Correct touch % (PRL DAS)	Group	0.78	1,43.5	0.38	0.38	Control; MS	65.9 ± 0.6; 64.9 ± 0.7	65.9 ± 0.6; 64.9 ± 0.6

	Sex	4.13	1,44.1	0.048	0.047	Female; Male	64.3 ± 0.7; 66.4 ± 0.6	64.4 ± 0.7; 66.4 ± 0.6
	Baseline	9.90	1,51.4	0.003	0.003			
	G×S	2.85	1,42.4	0.098	0.096			
	Group×SD	6.87	1,89.4	0.010	0.003			
	Sex×SD	0.00	1,87.8	0.95	0.93			
	G×S×SD	0.13	1,87.4	0.72	0.66			
	Stress day	1.88	1,90.3	0.17				
Reversal count (PRL DAS)	Group	0.27	1,43.6	0.61	0.61	Control; MS	3.45 ± 0.28; 3.23 ± 0.23	3.45 ± 0.24; 3.23 ± 0.25
	Sex	0.66	1,43.9	0.42	0.42	Female; Male	3.47 ± 0.32; 3.20 ± 0.19	3.48 ± 0.27; 3.20 ± 0.23
	Baseline	10.17	1,45.3	0.003	0.003			
	G×S	0.31	1,42.7	0.58	0.58			
	Group×SD	1.43	1,84.4	0.23	0.22			
	Sex×SD	0.27	1,84.1	0.61	0.60			
	G×S×SD	0.97	1,83.0	0.33	0.32			
	Stress day	0.18	1,86.5	0.67				
Trial count (PRL DAS)	Group	1.22	1,43.5	0.28	0.29	Control; MS	163.0 ± 5.2; 155.5 ± 4.2	163.1 ± 4.6; 155.6 ± 4.9
	Sex	4.27	1,43.1	0.045	0.045	Female; Male	167.1 ± 5.8; 151.4 ± 4.7	167.4 ± 5.5; 151.4 ± 4.7
	Baseline	12.19	1,40.7	0.001	4e-04			
	G×S	0.00	1,42.0	0.94	0.94			
	Group×SD	1.46	1,83.9	0.23	0.27			
	Sex×SD	0.00	1,83.8	0.96	0.96			
	G×S×SD	0.80	1,82.7	0.37	0.41			
	Stress day	0.04	1,86.1	0.84				
PR4 Breakpoint	Group	1.09	1,53.0	0.30	0.30	Control; MS	62.3 ± 2.2; 65.0 ± 1.7	62.3 ± 1.9; 65.0 ± 1.9
	Sex	1.14	1,53.0	0.29	0.29	Female; Male	62.2 ± 1.9; 65.1 ± 1.9	62.2 ± 2.0; 65.1 ± 1.8
	G×S	0.04	1,52.0	0.84	0.84			
	Group×Sess.	0.84	1,445.0	0.36	0.52			
	Sex×Sess.	1.10	1,445.0	0.29	0.45			
	G×S×Sess.	0.93	1,444.0	0.34	0.49			
	Session	28.77	1,447.0	1e-07				
PR8 Breakpoint	Group	0.01	1,53.0	0.92	0.92	Control; MS	82.2 ± 2.5; 81.7 ± 2.7	82.2 ± 2.5; 81.7 ± 2.6
	Sex	0.19	1,53.0	0.66	0.66	Female; Male	81.2 ± 2.6; 82.7 ± 2.5	81.1 ± 2.6; 82.7 ± 2.5
	G×S	0.05	1,52.0	0.82	0.82			
	Group×Sess.	0.97	1,442.2	0.33	0.43			
	Sex×Sess.	0.07	1,442.3	0.79	0.84			
	G×S×Sess.	1.31	1,441.3	0.25	0.35			
	Session	1.81	1,444.3	0.18				

PR16 Breakpoint	Group	1.48	1,52.0	0.23	0.23	Control; MS	87.9 ± 4.6; 95.0 ± 3.9	87.9 ± 4.3; 95.0 ± 4.3
	Sex	0.37	1,52.0	0.54	0.55	Female; Male	93.3 ± 3.9; 89.6 ± 4.5	93.3 ± 4.5; 89.6 ± 4.1
	G×S	0.04	1,51.0	0.85	0.85			
	Group×Sess.	0.30	1,437.0	0.58	0.63			
	Sex×Sess.	0.01	1,437.0	0.91	0.92			
	G×S×Sess.	0.27	1,436.0	0.60	0.64			
	Session	29.79	1,439.0	8e-08				
FR1 STC	Group	0.27	1,53.0	0.60	0.61			
	Sex	16.01	1,53.0	2e-04	0			
	G×S	0.05	1,52.0	0.83	0.83			
FR5 STC	Group	0.96	1,53.0	0.33	0.34			
	Sex	5.40	1,53.0	0.024	0.024			
	G×S	0.04	1,52.0	0.84	0.84			
TTB STC	Group	0.26	1,45.0	0.61	0.63			
	Sex	14.34	1,45.0	4e-04	1e-04			
	G×S	0.37	1,44.0	0.55	0.56			
DRL STC	Group	0.40	1,45.0	0.53	0.53			
	Sex	4.14	1,45.0	0.048	0.046			
	G×S	0.35	1,44.0	0.56	0.56			
PRL SC (BAS)	Group	3.06	1,45.0	0.087	0.089			
	Sex	6.40	1,45.0	0.015	0.015			
	G×S	0.78	1,44.0	0.38	0.40			
Weight (BAS)	Group	0.39	1,54.0	0.53	0.53	Control; MS	274.2 ± 3.2; 277.1 ± 3.2	274.2 ± 3.2; 277.1 ± 3.1
	Sex	1052.24	1,54.0	5e-37	0	Female; Male	199.3 ± 2.9; 352.0 ± 3.5	199.2 ± 3.2; 352.0 ± 3.1
	G×S	0.04	1,53.0	0.84	0.85			
	Group:Age	1.41	34,1632.2	0.059	0.21			
	Sex:Age	558.55	34,1632.2	0	0			
	G×S:Age	0.59	34,1598.2	0.97	0.74			
	Age	236.79	34,1702.2	0				
Weight (DAS)	Group	3.67	1,50.0	0.061	0.062	Control; MS	401.5 ± 2.1; 396.4 ± 2.4	401.6 ± 2.3; 396.3 ± 2.2
	Sex	2.29	1,49.6	0.14	0.14	Female; Male	409.3 ± 13.4; 388.6 ± 11.2	410.2 ± 8.5; 387.7 ± 6.7
	Baseline	396.72	1,48.6	5e-25	0			
	G×S	0.43	1,49.0	0.52	0.52			
	Group×SD	0.11	1,279.9	0.74	0.82			
	Sex×SD	9.65	1,279.9	0.002	0.039			
	SD×Baseline	16.39	1,278.9	7e-05	0.011			
	G×S×SD	2.16	1,279.0	0.14	0.33			

	Stress day	5.33	1,283.0	0.022				
Sucrose pref. (%) (BAS)	Group	0.94	1,52.9	0.34	0.34	Control; MS	79.6 ± 1.2; 77.9 ± 1.4	79.6 ± 1.4; 77.9 ± 1.4
	Sex	0.69	1,53.0	0.41	0.41	Female; Male	79.6 ± 1.2; 78.0 ± 1.4	79.6 ± 1.4; 77.9 ± 1.3
	G×S	1.24	1,52.0	0.27	0.26			
	Group×C	0.14	2,105.3	0.87	0.84			
	Sex×C	0.18	2,105.4	0.83	0.80			
	G×S×C	2.11	2,103.3	0.13	0.13			
	C	99.63	2,109.3	2e-25				
Sucrose pref. (%) (DAS)	Group	2.68	1,48	0.11		Control; MS		75.5 ± 1.7; 79.7 ± 1.7
	Sex	2.20	1,48	0.14		Female; Male		79.5 ± 1.9; 75.7 ± 1.6
	Baseline	1.96	1,48	0.17				
	G×S	0.67	1,48	0.42				
EPM Time in open arms (%)	Group	0.32	1,53	0.58		Control; MS		41.3 ± 3.1; 39.0 ± 3.1
	Sex	13.72	1,53	5e-04		Female; Male		48.2 ± 3.2; 32.0 ± 3.0
	G×S	0.31	1,53	0.58				
NPT Time in novel area (%)	Group	0.51	1,51	0.48		Control; MS		70.0 ± 1.7; 71.6 ± 1.7
	Sex	8.71	1,51	0.005		Female; Male		67.3 ± 1.7; 74.3 ± 1.6
	Familiar colour	5.67	1,51	0.021				
	Familiar texture	27.74	1,51	3e-06				
	G×S	1.30	1,51	0.26				
NRT Distance moved (m)	Group	0.00	1,53	0.96		Control; MS		174.2 ± 7.3; 174.7 ± 7.2
	Sex	1.52	1,53	0.22		Female; Male		180.8 ± 7.5; 168.1 ± 7.1
	G×S	0.03	1,53	0.87				
Amygdala vol.	Group	0.77	1,45.5	0.39	0.38	Control; MS	30.3 ± 0.1; 30.4 ± 0.1	30.3 ± 0.1; 30.4 ± 0.1
	Sex	16.68	1,74.5	1e-04	2e-04	Female; Male	29.9 ± 0.2; 30.7 ± 0.2	29.9 ± 0.2; 30.7 ± 0.1
	G×S	0.50	1,45.0	0.48	0.49			
	Group×T	4.72	2,66.0	0.012	0.018			
	Sex×T	2.02	2,76.3	0.14	0.15			
	G×S×T	1.13	2,63.9	0.33	0.33			
	Timepoint	495.89	2,78.5	3e-45				
	TBV	86.66	1,69.1	8e-14				
Cingulate cortex vol.	Group	0.28	1,45.0	0.60	0.60	Control; MS	27.4 ± 0.1; 27.6 ± 0.1	27.4 ± 0.1; 27.6 ± 0.1
	Sex	0.68	1,72.0	0.41	0.42	Female; Male	27.7 ± 0.2; 27.3 ± 0.2	27.7 ± 0.2; 27.3 ± 0.2
	G×S	0.05	1,44.5	0.82	0.82			
	Group×T	0.22	2,67.8	0.80	0.81			
	Sex×T	0.78	2,77.8	0.46	0.49			
	G×S×T	0.51	2,66.6	0.60	0.62			

	Timepoint	266.45	2,75.0	8e-35				
	TBV	63.75	1,75.6	1e-11				
Hippocampus vol.	Group	0.21	1,46.2	0.65	0.64	Control;<br>MS	83.0 ± 0.2;<br>82.9 ± 0.2	83.0 ± 0.2;<br>82.9 ± 0.2
	Sex	0.44	1,81.6	0.51	0.52	Female;<br>Male	82.8 ± 0.3;<br>83.0 ± 0.2	82.8 ± 0.3;<br>83.1 ± 0.3
	G×S	0.00	1,45.6	0.97	0.97			
	Group×T	0.07	2,63.7	0.94	0.94			
	Sex×T	0.02	2,74.3	0.98	0.98			
	G×S×T	0.11	2,62.4	0.90	0.90			
	Timepoint	1405.93	2,78.2	5e-62				
	TBV	143.10	1,85.8	6e-20				
Insula vol.	Group	0.00	1,44.0	0.96	0.96	Control;<br>MS	35.3 ± 0.1;<br>35.4 ± 0.1	35.3 ± 0.1;<br>35.4 ± 0.1
	Sex	0.20	1,69.3	0.65	0.65	Female;<br>Male	35.2 ± 0.2;<br>35.5 ± 0.2	35.2 ± 0.2;<br>35.5 ± 0.2
	G×S	0.18	1,43.9	0.67	0.67			
	Group×T	0.53	2,69.5	0.59	0.57			
	Sex×T	1.61	2,79.3	0.21	0.18			
	G×S×T	0.83	2,68.2	0.44	0.42			
	Timepoint	427.07	2,79.9	2e-43				
	TBV	155.15	1,64.4	8e-19				
Nucleus accumbens vol.	Group	0.46	1,45.5	0.50	0.51	Control;<br>MS	11.0 ± 0.0;<br>11.1 ± 0.1	11.0 ± 0;<br>11.1 ± 0
	Sex	0.36	1,75.4	0.55	0.54	Female;<br>Male	11.0 ± 0;<br>11.0 ± 0	11.0 ± 0;<br>11.0 ± 0
	G×S	0.00	1,45.0	0.96	0.96			
	Group×T	0.01	2,66.1	0.99	0.99			
	Sex×T	1.35	2,76.4	0.27	0.31			
	G×S×T	0.08	2,65.0	0.93	0.93			
	Timepoint	840.18	2,74.9	5e-52				
	TBV	54.89	1,75.0	2e-10				
Dorsal striatum vol.	Group	0.23	1,46.5	0.63	0.63	Control;<br>MS	66.9 ± 0.2;<br>66.7 ± 0.2	66.9 ± 0.2;<br>66.7 ± 0.2
	Sex	0.50	1,83.4	0.48	0.49	Female;<br>Male	67.1 ± 0.3;<br>66.5 ± 0.2	67.2 ± 0.3;<br>66.4 ± 0.3
	G×S	0.13	1,45.9	0.72	0.72			
	Group×T	2.40	2,62.3	0.099	0.11			
	Sex×T	3.26	2,72.8	0.044	0.044			
	G×S×T	0.13	2,60.8	0.88	0.89			
	Timepoint	2678.47	2,76.8	9e-72				
	TBV	126.50	1,84.6	2e-18				

Figure S6. Test statistics from all models, including for terms not related to hypotheses of interest (e.g. main effects of sex and time and their interactions). Estimated marginal means are also provided in relation to some predictors. Abbreviations (ordered alphabetically): ×C: × sucrose concentration (categorical); ×S: × sex; ×T: × timepoint; BAS: before adult stress; C: controls; DAS: during adult stress; DRL: deterministic reversal learning; EMMs: estimated marginal means; EPM: elevated plus maze; F:

females; FR1: fixed ratio 1; FR5: fixed ratio 5; M: males; ms: milliseconds; MS: repeated maternal separation; NPT: novelty preference test; NRT: novelty reactivity test; par.: parametric; per.: permutation test; PRL: probabilistic reversal learning; s: seconds; S: session; TBV: total brain volume (mean-centered within timepoint); TTA: touch training A; TTB: touch training B; SC: session count; SPT: sucrose preference test; STC: sessions to criterion.

References

1 Rohatgi A. *Webplotdigitizer: Version 4.5*, 2021. https://automeris.io/WebPlotDigitizer.

2 Clemens LE, Jansson EKH, Portal E, Riess O, Nguyen HP. A behavioral comparison of the common laboratory rat strains Lister Hooded, Lewis, Fischer 344 and Wistar in an automated homecage system. *Genes, brain, and behavior* 2014; **13**: 305–321.

3 Fell MJ, McDermott E, Sisodia G, Neill JC, Marshall KM. The relationship between starting body weight and weight gain in female hooded-lister rats. In.

4 Pellow S, Chopin P, File SE, Briley M. Validation of open:closed arm entries in an elevated plus-maze as a measure of anxiety in the rat. *Proceedings of the National Academy of Sciences* 1985; **14**: 149–167.

5 Belin D, Berson N, Balado E, Piazza PV, Deroche-Gamonet V. High-novelty-preference rats are predisposed to compulsive cocaine self-administration. *Neuropsychopharmacology official publication of the American College of Neuropsychopharmacology* 2011; **36**: 569–579.

6 Alfons A. *robustHD: Robust Methods for High-Dimensional Data*, 2019. https://CRAN.R-project.org/package=robustHD.

7 Leys C, Ley C, Klein O, Bernard P, Licata L. Detecting outliers: Do not use standard deviation around the mean, use absolute deviation around the median. *Journal of Experimental Social Psychology* 2013; **49**: 764–766.

8 Iglewicz B, Hoaglin DC. *How to detect and handle outliers.* The ASQC basic references in quality control statistical techniques, v.16. ASQC: Milwaukee, Wis., 1993.

9 Avants BB, Tustison NJ, Song G, Cook PA, Klein A, Gee JC. A reproducible evaluation of ANTs similarity metric performance in brain image registration. *NeuroImage* 2011; **54**: 2033–2044.

10 Tustison NJ, Cook PA, Klein A, Song G, Das SR, Duda JT *et al.* Large-scale evaluation of ANTs and FreeSurfer cortical thickness measurements. *NeuroImage* 2014; **99**: 166–179.

11 Jenkinson M, Beckmann CF, Behrens TEJ, Woolrich MW, Smith SM. FSL. *NeuroImage* 2012; **62**: 782–790.

12 Smith SM, Jenkinson M, Woolrich MW, Beckmann CF, Behrens TEJ, Johansen-Berg H *et al.* Advances in functional and structural MR image analysis and implementation as FSL. *NeuroImage* 2004; **23 Suppl 1**: S208-19.

13 Tournier J-D, Smith R, Raffelt D, Tabbara R, Dhollander T, Pietsch M *et al.* MRtrix3: A fast, flexible and open software framework for medical image processing and visualisation. *NeuroImage* 2019; **202**: 116137.

14 Yushkevich PA, Piven J, Hazlett HC, Smith RG, Ho S, Gee JC *et al.* User-guided 3D active contour segmentation of anatomical structures: significantly improved efficiency and reliability. *NeuroImage* 2006; **31**: 1116–1128.

15 Tustison NJ, Avants BB, Cook PA, Zheng Y, Egan A, Yushkevich PA *et al.* N4ITK: improved N3 bias correction. *IEEE transactions on medical imaging* 2010; **29**: 1310–1320.

16 Avants BB, Epstein CL, Grossman M, Gee JC. Symmetric diffeomorphic image registration with cross-correlation: evaluating automated labeling of elderly and neurodegenerative brain. *Medical image analysis* 2008; **12**: 26–41.

17 Liang X, Hsu L-M, Lu H, Sumiyoshi A, He Y, Yang Y. The Rich-Club Organization in Rat Functional Brain Network to Balance Between Communication Cost and Efficiency. *Cerebral cortex (New York, N.Y. 1991)* 2018; **28**: 924–935.

18 Valdés-Hernández PA, Sumiyoshi A, Nonaka H, Haga R, Aubert-Vásquez E, Ogawa T *et al.* An in vivo MRI Template Set for Morphometry, Tissue Segmentation, and fMRI Localization in Rats. *Frontiers in neuroinformatics* 2011; **5**: 26.

19 Calabrese E, Badea A, Watson C, Johnson GA. A quantitative magnetic resonance histology atlas of postnatal rat brain development with regional estimates of growth and variability. *NeuroImage* 2013; **71**: 196–206.

20 Schwarz AJ, Danckaert A, Reese T, Gozzi A, Paxinos G, Watson C *et al.* A stereotaxic MRI template set for the rat brain with tissue class distribution maps and co-registered anatomical atlas: application to pharmacological MRI. *NeuroImage* 2006; **32**: 538–550.

21 Paxinos G, Watson C. *The rat brain in stereotaxic coordinates*, 6th edn. Elsevier: Amsterdam, 2007.

22 Paxinos G, Watson C. *The rat brain in stereotaxic coordinates*, 4th edn. Academic: San Diego, London, 1998.

23 Rorden C, Brett M. Stereotaxic display of brain lesions. *Behavioural neurology* 2000; **12**: 191–200.

24 Calabrese E, Johnson GA, Watson C. An ontology-based segmentation scheme for tracking postnatal changes in the developing rodent brain with MRI. *NeuroImage* 2013; **67**: 375–384.

25 Wickham H. *ggplot2: Elegant Graphics for Data Analysis*. Springer-Verlag New York, 2016.

26 Wei T, Simko V. *R package "corrplot": Visualization of a Correlation Matrix*, 2017. https://github.com/taiyun/corrplot.

27 Pedersen TL. *patchwork: The Composer of Plots*, 2020. https://CRAN.R-project.org/package=patchwork.

28 Bates D, Mächler M, Bolker B, Walker S. Fitting Linear Mixed-Effects Models Using lme4. *Journal of Statistical Software* 2015; **67**: 1–48.

29 Fox J, Weisberg S. *An R Companion to Applied Regression*. Sage: Thousand Oaks CA, 2019.

30 Luke SG. Evaluating significance in linear mixed-effects models in R. *Behavior research methods* 2017; **49**: 1494–1502.

31 Lenth RV. *emmeans: Estimated Marginal Means, aka Least-Squares Means*, 2021. https://CRAN.R-project.org/package=emmeans.

32 Goode K, McClernon K, Zhao J, Zhang Y, Huo Y. *redres: Residuals and Diagnostic Plots for Mixed Models*, 2021. https://github.com/goodekat/redres.git.

33 Hartig F. *DHARMa: Residual Diagnostics for Hierarchical (Multi-Level / Mixed) Regression Models*, 2021. https://CRAN.R-project.org/package=DHARMa.

34 Buskirk TD, Willoughby LM, Tomazic TJ. Nonparametric Statistical Techniques. In Little TD (ed). *The Oxford handbook of quantitative methods*. Oxford library of psychology. Oxford University Press: New York, 2013.

35 LaFleur BJ, Greevy RA. Introduction to permutation and resampling-based hypothesis tests. *Journal of clinical child and adolescent psychology the official journal for the Society of Clinical Child and Adolescent Psychology, American Psychological Association, Division 53* 2009; **38**: 286–294.

36 Ludbrook J, Dudley H. Why Permutation Tests are Superior to t and F Tests in Biomedical Research. *The American Statistician* 1998; **52**: 127–132.

37 Winkler AM, Ridgway GR, Webster MA, Smith SM, Nichols TE. Permutation inference for the general linear model. *NeuroImage* 2014; **92**: 381–397.

38 Helwig NE. Bootstrap Confidence Intervals, 2017. http://users.stat.umn.edu/~helwig/notes/bootci-Notes.pdf (accessed 24-Jun-21).

39 Hesterberg T. *What Teachers Should Know about the Bootstrap: Resampling in the Undergraduate Statistics Curriculum*, 2014.

40 Hesterberg T. *resample: Resampling Functions*, 2015. https://CRAN.R-project.org/package=resample.

41 Senn S. Change from baseline and analysis of covariance revisited. *Statistics in medicine* 2006; **25**: 4334–4344.

42 Landau S, Emsley R, Dunn G. Beyond total treatment effects in randomised controlled trials: Baseline measurement of intermediate outcomes needed to reduce confounding in mediation investigations. *Clinical trials (London, England)* 2018; **15**: 247–256.

43 Wan F. Statistical analysis of two arm randomized pre-post designs with one post-treatment measurement. *BMC medical research methodology* 2021; **21**: 150.
